# IFN-α Regulates Blimp-1 Expression via miR-23a and miR-125b in Both Monocytes-Derived DC and pDC

**DOI:** 10.1371/journal.pone.0072833

**Published:** 2013-08-16

**Authors:** Stefania Parlato, Roberto Bruni, Paola Fragapane, Debora Salerno, Cinzia Marcantonio, Paola Borghi, Paola Tataseo, Anna Rita Ciccaglione, Carlo Presutti, Giulia Romagnoli, Irene Bozzoni, Filippo Belardelli, Lucia Gabriele

**Affiliations:** 1 Department of Hematology, Oncology and Molecular Medicine, Istituto Superiore di Sanità, Rome, Italy; 2 Department of Infectious, Parasitic and Immune-Mediated Diseases, Istituto Superiore di Sanità, Rome, Italy; 3 Institute of Molecular Biology and Pathology, Consiglio Nazionale delle Ricerche, Rome, Italy; 4 Transfusional Medicine and Molecular Biology Laboratory, ASL, Avezzano-Sulmona, Sulmona, Italy; 5 Department of Genetics and Molecular Biology, Sapienza University, Rome, Italy; University Medical Center Freiburg, Germany

## Abstract

Type I interferon (IFN-I) have emerged as crucial mediators of cellular signals controlling DC differentiation and function. Human DC differentiated from monocytes in the presence of IFN-α (IFN-α DC) show a partially mature phenotype and a special capability of stimulating CD4+ T cell and cross-priming CD8+ T cells. Likewise, plasmacytoid DC (pDC) are blood DC highly specialized in the production of IFN-α in response to viruses and other danger signals, whose functional features may be shaped by IFN-I. Here, we investigated the molecular mechanisms stimulated by IFN-α in driving human monocyte-derived DC differentiation and performed parallel studies on peripheral unstimulated and IFN-α-treated pDC. A specific miRNA signature was induced in IFN-α DC and selected miRNAs, among which miR-23a and miR-125b, proved to be negatively associated with up-modulation of Blimp-1 occurring during IFN-α-driven DC differentiation. Of note, monocyte-derived IFN-α DC and *in vitro* IFN-α-treated pDC shared a restricted pattern of miRNAs regulating Blimp-1 expression as well as some similar phenotypic, molecular and functional hallmarks, supporting the existence of a potential relationship between these DC populations. On the whole, these data uncover a new role of Blimp-1 in human DC differentiation driven by IFN-α and identify Blimp-1 as an IFN-α-mediated key regulator potentially accounting for shared functional features between IFN-α DC and pDC.

## Introduction

IFN-I are pleiotropic cytokines that also coordinate innate and adaptive immune responses. In the past few years, IFN-I have emerged as crucial mediators of cellular signals controlling DC function [1]. Our group as well as other laboratories reported that human DC differentiated from peripheral monocytes in the presence of IFN-α (IFN-α DC) represent highly activated DC capable of efficiently inducing CD8+ T cells against both viral and tumor antigens [2–4]. IFN-α DC have been shown to retain phagocytic activity, in spite of their partial mature phenotype, and to own a special capability of stimulating CD4+ T helper lymphocytes and inducing cross-priming of CD8+ T cells [5]. Of note, IFN-α DC have been reported to exhibit a combined phenotype of plasmacytoid DC (pDC) and conventional DC (cDC) associated with natural killer (NK) cell characteristics [6]. pDC are blood DC highly specialized in the production of IFN-α in response to viruses and other danger signals, whose functional features may be shaped by exposure to IFN-I. In particular, it has been recently reported that IFN-α influences the development of pDC, since its systemic delivery stimulates production of these cells at bone-marrow level [7]. pDC, known to be implicated in the immune responses occurring during many diseases including autoimmunity and cancer [8,9], share similarities with several different cell types among which cDC and B lymphocytes dominate. The prominent lymphoid features of pDC may be due to the activity of lymphoid transcription factors that may orchestrate pDC-specific gene expression shared with B cells [10]. To date, the mechanisms underlying the ability of IFN-α to confer DC with unique functions as well as those regulating IFN-I influence on pDC development and function remain unclear.

B lymphocyte-induced maturation protein-1 (Blimp-1), encoded by the *PRDM-1* gene, was first discovered as a transcriptional repressor of the IFN-β promoter [11]. Blimp-1 is a master regulator of effector and memory differentiation in B cells as well as in CD4+ and CD8+ T cells [12,13]. Of interest, this regulator governs transcriptional networks in B lymphocytes and germ cells resulting in stable silencing of pivotal transcription factors such as PAX5 and CIITA, that shift the gene expression program towards terminal plasma cell differentiation [14]. Recently, conditional knockout of Blimp-1 in hematopoietic lineages revealed its role in homeostatic development and functional maturation of cDC [15]. Moreover, during the development and function of myeloid cells Blimp-1 has been shown to establish a cross-talk with IRF8, a well-known master regulator of pDC, suggesting their coordinate role in cytokine production and antigen presentation [16]. Therefore, investigating the expression and the activity of Blimp-1 in pDC may shed light on mechanisms behind their lineage affiliation and function.

MicroRNAs (miRNAs) are key regulators modulating gene expression, thus influencing cell fate and function [17]. In the immune system, miRNAs are active during hematopoietic development and cell subset differentiation and control effector cell functions. Likewise, DC development from myeloid precursors and differentiation into specialized subsets are also regulated by miRNAs [18]. DC differentiation from human monocytes in the presence of IL-4 is characterized by differential expression of 20 miRNAs controlling target genes implicated in this process [19]. Recently, miR-221 and miR-155 have been described to play a role during DC maturation, apoptosis and IL-12 production [20]. Moreover, IFN-I has been shown to regulate TLR7-induced pDC activation via regulation of miR-155 and miR-155* [21]. Distinct miRNA expression signatures were also reported to characterize pDC as closely related to B lymphocytes more than cDC [22]. Of interest, selected miRNAs, such as miR-125b, have been shown to target PRDM1/Blimp-1 gene [23]. Therefore, although miRNAs have emerged as critical players in DC subset differentiation and specialization as well as in DC maturation and function, how their expression may impact the rapid regulation of gene expression controlling DC activity is poorly understood.

In this study, we investigated the molecular mechanisms stimulated by IFN-α in driving human monocytes-derived DC differentiation. The results reveal that a specific miRNA signature is selectively modulated in IFN-α DC as compared to conventional IL-4 DC. Of interest, the down-modulation of a selected panel of IFN-α-driven miRNAs, among which miR-23a and miR-125b are the major players, tightly associates with up-modulation of Blimp-1 occurring during DC differentiation in these experimental conditions. Importantly, IFN-α DC and IFN-α-treated peripheral pDC display the same pattern of miRNAs controlling Blimp-1 expression as well as some similar phenotypic and molecular features. Moreover, these two cell populations were able to produce IFN-I following NDV infection. These findings underscore novel molecular mechanisms by which IFN-α drives differentiation of human DC and identify Blimp-1 as an IFN-α-driven key regulator potentially accounting for shared functional features between IFN-α DC and pDC.

## Materials and Methods

### Ethic Statement

Peripheral blood mononuclear cells (PBMC) utilized in this study derived from buffy coats obtained from healthy blood donors, as anonymously provided by the Immunohematology and Transfusional Center of Policlinico Umberto I, Sapienza University, Rome. Written informed consent for the use of buffy coats for research purposes was obtained from blood donors by the Transfusional Center.

### Cell Culture

PBMC were isolated by density-gradient centrifugation (Lymphoprep). Monocyte-derived DC were generated from CD14+ monocytes (CD14 Microbeads, Miltenyi Biotec) cultured at concentration of 2x10^6^ cells/ml for 3 days in RPMI containing 10% LPS-free FCS in presence of 500 U/ml GM-CSF (PeproTech) alone or in combination with either 250 U/ml IL-4 (R&D Systems) (IL-4 DC) or natural IFN-α (Alfaferone; AlfaWasserman) at concentration of 10,000 U/ml (IFN-α DC). In some experiments IL-4 DC were treated with 10 ng/ml TNFα for 24 hours. Peripheral pDC (CD123+, CD11c-, Lineage-) were isolated from total PBMC by negative immunoselection (Plasmacytoid dendritic cell isolation kit, Miltenyi Biotec) to a purity of more than 90%, as assessed by flow cytometry analysis. pDC were seeded at a density of 10^5^ cells/200 µl of complete RPMI 1640 medium and treated or not with 10,000 U/ml IFN-α for 24 hours. HeLa cell line was maintained in D-MEM medium supplemented with penicillin/streptomycin solution, L-glutamine and 10% FCS.

### Immunophenotypic Analysis

Cells were analyzed by FACSCalibur flow cytometer (BD Biosciences) after staining with specific antibodies: -CD123, -CD1a, -CD11c, -CD14, -CD3, -CD19, -CD56 (all from BD Biosciences); anti-BDCA-1, -BDCA-2, and -BDCA-4 (Miltenyi Biotec).

### MiRNA Quantification

Total RNA was extracted from cells by miRNeasy mini kit (Qiagen). RNA concentration, purity and integrity were determined by using NanoDrop 1000 (Thermo, Fisher Scientific) and 2100 Bioanalyzer (Agilent Technologies). MiRNA quantification was carried out by real-time PCR with TaqMan^®^ MicroRNA Assays, according to manifacturer’s instructions (Applied Biosystems). Fold induction was calculated by 2^-ΔCt^ method, using GM-CSF-treated monocytes as calibrators (control), as previously reported [24].

### Prediction of Genes Targeted by miRNAs

Putative gene targets of miRNAs were predicted by means of miRGator program (available at http://genome.ewha.ac.kr/miRGator/miRNAexpression.html) combining gene predictions by TargetScan, miRanda and PicTar softwares. To avoid loss of potential targets, a relaxed option was selected. Gene lists were analyzed by Excel program to search for genes targeted by multiple miRNAs.

### Quantitative Real-Time PCR

Total RNA was extracted from cells by RNeasy mini kit (Qiagen). The RNA concentration and integrity analysis was performed as previously described in “MicroRNA quantification” section. Total RNA, was reverse transcribed into cDNA (cDNA synthesis kit, Bioline) and analyzed by *qRT-PCR* (SensiMix Plus SYBR kit, Quantace) using the following oligonucleotides: BLIMP-1F (5’-GTGTCAGAACGGGATGAAC-3’), BLIMP-1-R (5’-TGTTAGAACGGTAGAGGTCC-3’), CD2AP-F (5’-AGGAATGTTCCCTGACAATTTCG-3’), CD2AP-R (5’-GCTGGAAGTCCATATAGGTGCTT-3’), IRF-8-F (5’-GCTGATCAAGGAGCCTTCTG-3’), IRF-8-R (5’-ACCAGTCTGGAAGGAGCTGA-3’), TLR7-F (5’-ACAAGATGCCTTCCAGTTGC-3’), TLR7-R (5’-ACATCTGTGGCCAGGTAAGG-3’), TLR9-F (5’-GTGCCCCACTTCTCCATG-3’), TLR9-R (5’-GTGCCCCACTTCTCCATG-3’), β-act-F (5’-GATCCGCCGCCCGTCCACA-3’), β-act-R (5’-GACGATGCCGTGCTCGATG-3’). Amplification was performed in 7500 real-time PCR System (Applied biosystems). The relative expression level of each mRNA was normalized to β-actin using the comparative 2^-ΔCt^ method.

### Western Blot Analysis

Cells were lysed in ice-cold extraction buffer containing 50 mmol/l Tris-HCl (pH 8), 1 mmol/l EGTA, 150 mM NaCl, 10% glycerol, 1.5 mM MgCl2, 1% Triton, 50 mM NaF and a protease inhibitor cocktail (Roche Applied Science). After centrifugation, 30 µg of protein extracts contained in supernatant fraction were loaded onto a NuPAGE Bis-Tris Minigel 4/12% 1 mm (Invitrogen) and transferred to membranes (Protran; Schleicher & Schuell BioScience). Blots were probed with mouse anti-human Blimp-1 IgG1 (Novus Biologicals). Protein levels were verified by immunoblotting with mouse anti-human Gapdh IgG1 (Santa Cruz). Immunoreactive bands were detected with goat anti-mouse IgG horseradish peroxidase-conjugated secondary antibody and visualized by SuperSignal chemiluminescent substrate (Pierce). Densitometry analysis was performed by using ImageJ software (rsb.info.nih.gov/ij).

### HeLa Transfection

HeLa cells were transfected with miR-23a and miR-125b or with both miRNAs using lipofectamine according to the manufacturer’s instructions (Invitrogen). The pU1-23a plasmid was generated by cloning a genomic PCR fragment from a plasmid containing 23a cluster (gift from Dr Neeru Saini) into the pSP65-U1 cassette plasmid [25,26]. The plasmid containing miR-125b was a gift from Dr Ubaldo Gioia. Total RNA from transfected HeLa cells was extracted and analyzed by Northern blot as previously described [27]. HeLa cells were also transfected with a PRDM-1-unrelated miRNA (miR-1) and a synthetic miRNA (Exiqon), used as controls. In some experiments, after 24 hours from transfection cells were treated for further 24 or 48 hours with different doses of IFN-α and analyzed for the expression of Blimp-1.

### In Vitro Stimulation of DC and IFN Bioassay

3 x 10^5^ monocyte-derived IFN-α DC, IL-4 DC and peripheral pDC were infected with Newcastle disease virus (NDV; 576 UE/ml) for 1 hour at 37°C, 5% CO2, then washed and cultured in 300 µl/well of a 48 well-plate for 18 hours, after which the supernatant was harvested. 50 µl of each sample was assayed for IFN-I biological activity by measuring its ability to confer resistance to vescicular stomatitis virus (VSV) infection upon L929 cells as described elsewhere [28]. Each IFN unit, as expressed in the text, represents 4 IU.

### Statistics

Statistical analyses were performed using Wilcoxon test for paired samples (two tailed) and Mann–Whitney test for unpaired samples using STATA software, version 8.0. *p*-value below 0.05 was considered significant.

## Results

### IFN-α induces a specific miRNA signature during DC differentiation and modulates miRNAs targeting Blimp-*1*

**Figure 1:**
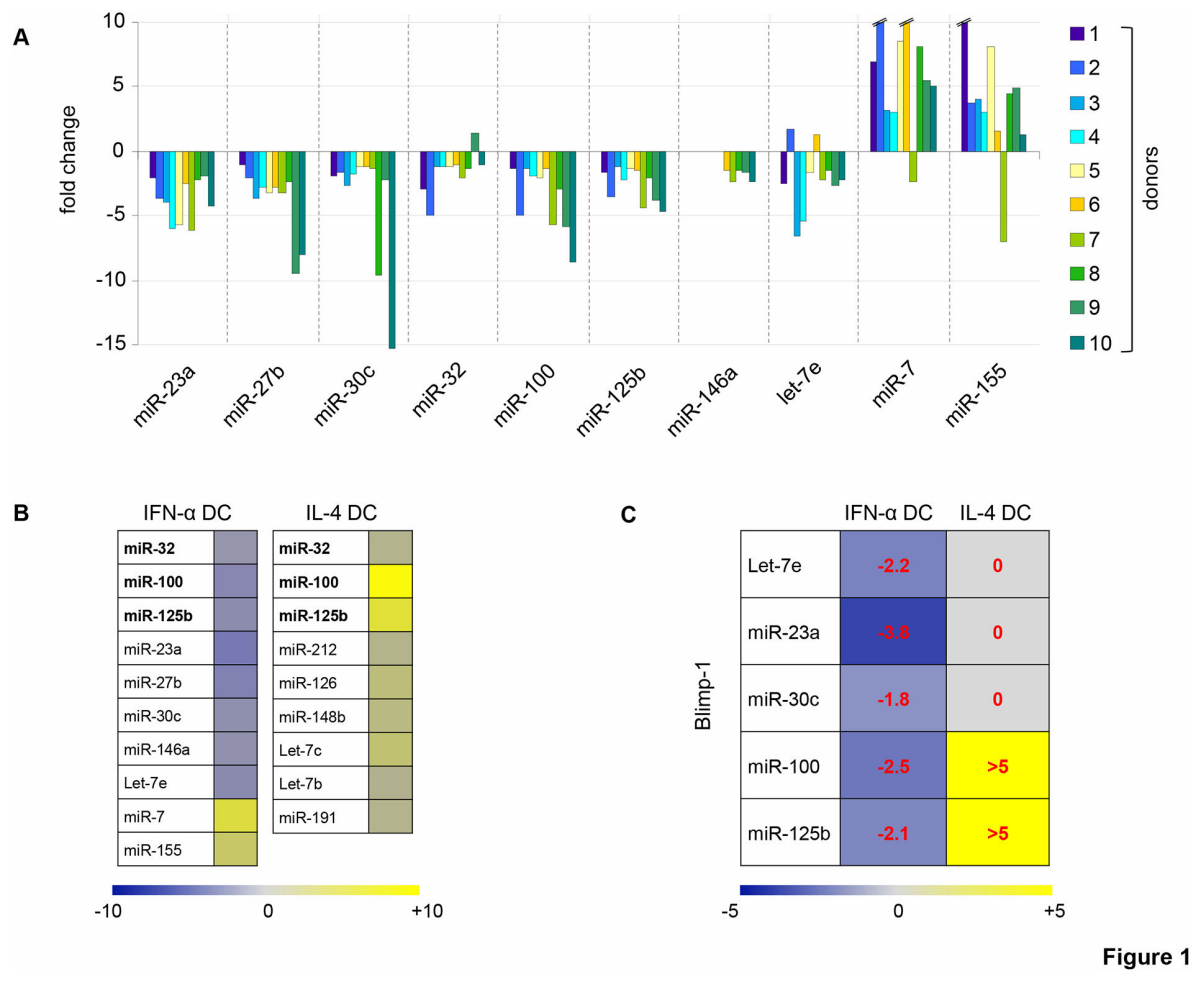
IFN-α induces a specific pattern of miRNAs during DC differentiation: *PRDM-1* is a miRNA target gene. **A**. Fold induction of specific miRNAs in DC differentiated from monocytes after IFN-α treatment with respect to monocytes treated with GM-CSF alone. Each miRNA was analyzed by qRT-PCR on 10 different donors, except miR-146a, whose data were available only for 5 donors (donor 6 to 10). Broken lines indicate out of range values. **B**. miRNAs found to be up-regulated or down-regulated, with respect GM-CSF-treated monocytes, in DC differentiated in the presence of IFN-α or IL-4. Color intensities and numbers indicate the relative median of fold-change values. Fold-change threshold was fixed at ± 1.2 in at least 8 out of 10 investigated donors for IFN-α DC and in at least 4 out of 5 donors for IL-4 DC and for miR-146a in IFN-α DC. Bold type represents miRNAs whose expression changed in both IFN-α DC and IL-4 DC, but in opposite direction. **C**. Expression of miRNAs whose target site is predicted in the 3’ UTR of the Blimp-1-coding *PRDM-1* gene in DC differentiated with IFN-α or IL-4. Median fold-changes are indicated in each colored box.

IFN-α DC are highly activated DC, obtained by one-step differentiation of GM-CSF-treated human monocytes in the presence of IFN-α. Indeed, the characterization of the phenotype and functions of IFN-α DC has led to the concept that these cells, as opposite to conventional IL-4 DC, can resemble naturally occurring DC, rapidly generated from monocytes in response to danger signals such as infection [4,29]. In this study, we sought to further dissect the effects of IFN-α on DC development by investigating the miRNA signature in IFN-α DC as compared to that of IL-4 DC. Thus, we evaluated the expression pattern of a set of 30 miRNAs in IFN-α DC and IL-4 DC generated from 5 different healthy donors (Table S1). We detected a defined panel of 10 miRNAs characterizing the IFN-α treatment that we further assessed in DC generated from 5 additional healthy donors: miR-100, miR-125b, miR-32, miR-23a, miR-30c, miR-27b, miR-146a and let-7e resulted to be significantly down-modulated, whereas miR-7 and miR-155 were markedly up-modulated (Figure 1A). Of interest, the pattern of miRNAs in IFN-α DC was quite different from that observed in IL-4 DC, since these populations shared only the modulation of miR-100, miR-125b and miR-32, but in an opposite manner. Moreover, relatively few miRNAs among those tested exhibited a significant modulation in IL-4 DC (Figure 1B). Table S2 details the fold change and *p*-value of the differentially regulated miRNAs.

Since we found strongly modulated 10 miRNAs by IFN-α during DC differentiation, we sought to identify the genes targeted by these miRNAs by using a computational approach which relies on miRGator, a miRNA target prediction program [30] (Table S3). We found *PRDM-1* gene, encoding Blimp-1, predicted to be the target gene of 5 out of 10 miRNAs regulated in IFN-α DC: miR-23a, miR-30c, miR-100, miR-125b and let-7e (Figure 1C). Of interest, all miRNAs predicted to regulate Blimp-1 expression were concordantly down-modulated by IFN-α; on the contrary, during DC differentiation driven by IL-4, miR-100 and miR-125b resulted up-modulated, whereas miR-23a, miR-30c and let-7e were not differentially expressed compared to the untreated control (Figure 1C). Therefore, IFN-α DC show a distinctive miRNA profile characterized by the down-regulation of miRNAs targeting Blimp-1.

### Blimp-*1* is modulated by IFN-α during monocyte-derived DC differentiation via miR-23a and miR-125b

**Figure 2:**
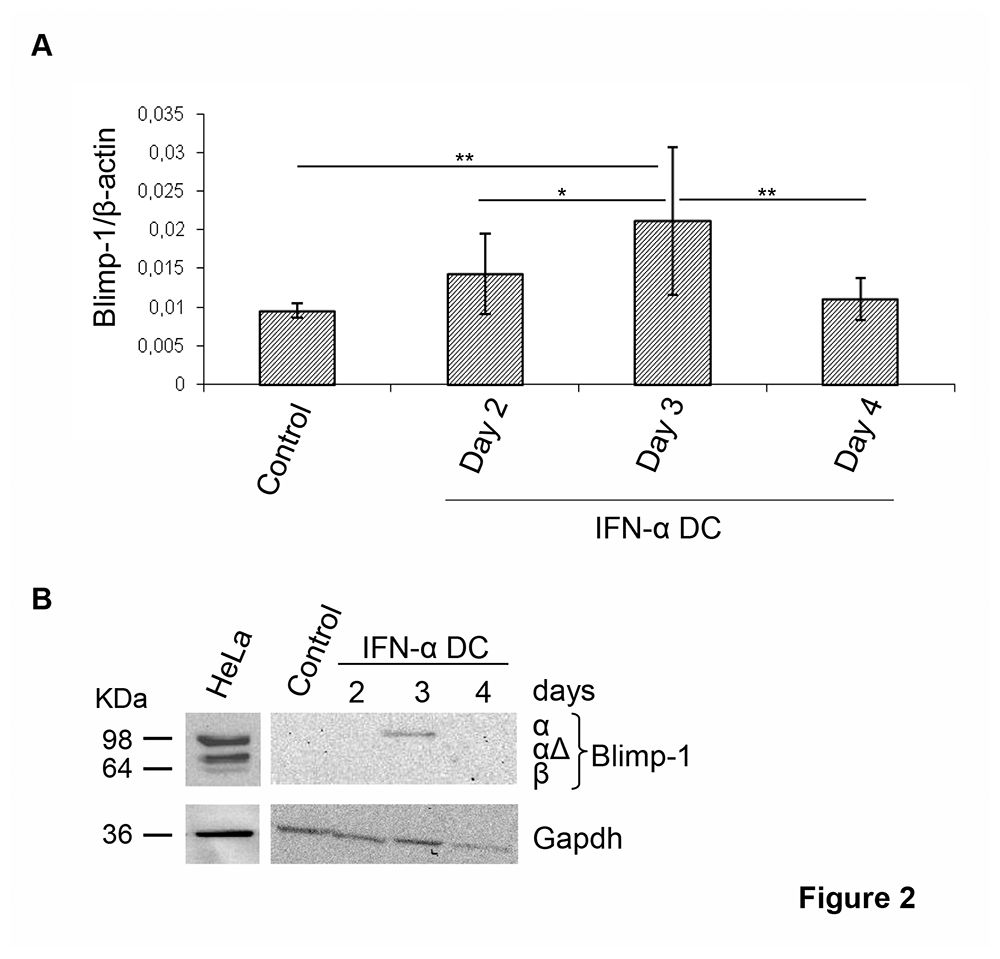
IFN-α stimulates the expression of Blimp-1. **A**. Kinetics of Blimp-1 expression in IFN-α DC was analyzed by qRT-PCR with respect to freshly isolated monocytes. Normalized data represent the mean ± SD of 4 independent experiments. Wilcoxon test was performed: *p= 0.0034; **p= 0.0005. **B**. Blimp-1 protein expression analyzed by western blot from day 2 to day 4 in IFN-α DC and monocytes. HeLa cell line was used as control of expression. Data from 1 representative experiment out of 3 are shown.

**Figure 3:**
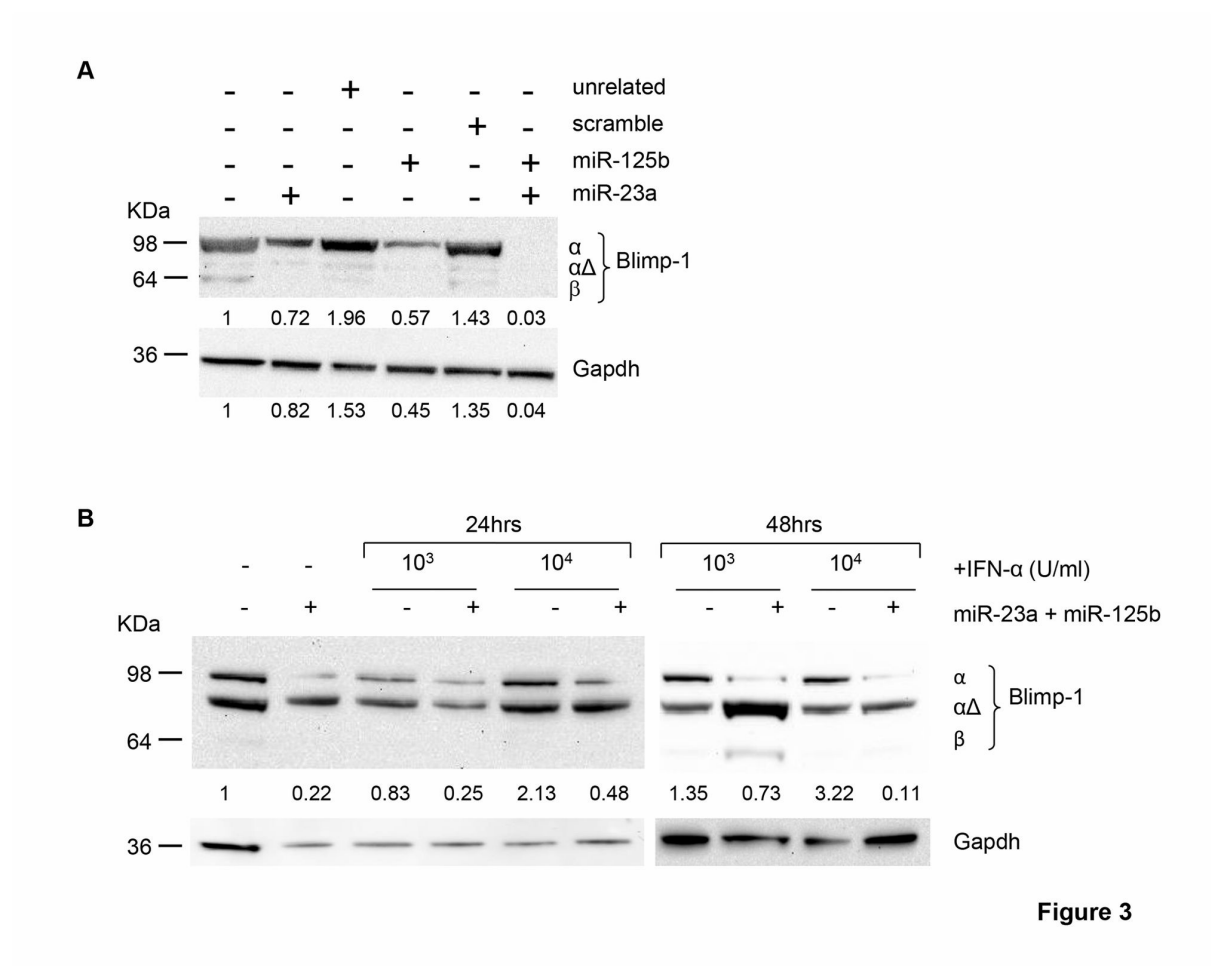
IFN-α-driven Blimp-1 expression is under the control of miR-23a and miR-125b. **A**. Blimp-1α protein reduction upon transfection of miR-23a and miR-125b in HeLa cells, measured by western blot. As a control, HeLa cells transfected with empty plasmid or unrelated miRNAs (unrelated1: miR-1, unrelated2: miR-159sense) were used. Intensities of bands were measured and numbers indicate the values expressed as fold induction with respect to Gapdh. 1 representative experiment out of 3 is shown. **B**. Blimp-1 expression in HeLa cells transfected or not with single or combined miR-23a and miR-125b and treated for 24 or 48 hours with the indicated doses of IFN-α. HeLa cells transfected with empty plasmid were used as a control. Numbers indicate Blimp-1α isoform quantification expressed as fold induction with respect to Gapdh. 1 representative experiment out of 3 is shown.

Given that the functional significance of the coordinated down-modulation of multiple miRNAs targeting one gene predicts a tight regulation of its activity [31], we focused on the evaluation of Blimp-1 expression in DC during IFN-α-driven differentiation. Consistently with miRNA data, we found that the induction of Blimp-1 by IFN-α during DC commitment was time-dependent, because the peak of transcript levels occurred lately during the differentiation process at day 3 of culture, when IFN-α DC fully express DC activation markers [32] (Figure 2A). Of interest, Blimp-1 transcripts were slightly increased after 2 days of *in vitro* differentiation and rapidly declined at day 4 of culture (Figure 2A). Then, we investigated the protein levels of Blimp-1. In this regard, three distinct Blimp-1 isoforms have been described: the active full-length α isoform (Blimp-1α), the β isoform (Blimp-1β) with a damped activity and the intermediate-sized Blimp-1αΔ isoform, reported to interfere negatively with Blimp-1α [33,34]. Notably, only Blimp-1α isoform was clearly detected in extracts derived from IFN-α DC at day 3 of differentiation (Figure 2B). Conversely, IL4-DC, that did not display the modulation of miRNAs targeting *PRDM-1* gene, were found to express barely levels of Blimp-1 transcripts comparable to those of monocytes. Moreover, treatment of these cells with a maturation stimulus such as TNF-α did not induce further Blimp-1 transcription (Figure S1A). Remarkably, the uniqueness of IFN-α-induced miRNA signature targeting Blimp-1 was confirmed by the further up-regulation of miR-125b and the absence of miR-23a modulation in IL4-DC treated with TNF-α (Figure S1B). Of interest, among the IFN-α-regulated miRNAs targeting Blimp-1, miR-23a and miR-125b resulted the only ones recently reported to be implicated in B cell development [35,36]. Therefore, to further investigate whether during IFN-α-driven cellular differentiation the induction of Blimp-1 was mediated mainly by the activity of these two miRNAs, we devised a strategy to track the expression of Blimp-1 in HeLa cells following over-expression of single or combined miRNAs, in presence or absence of IFN-α. It is noteworthy to mention that HeLa cells in use in our laboratory exhibited stable basal levels of Blimp-1α protein as well as almost undetectable expression of the inactive Blimp-1β isoform and, upon several culture passages, they up-regulated Blimp-1αΔ (Figure 3A and B). By transfecting HeLa cells, we over-expressed miR-23a and miR-125b, alone or in combination (Figure S2). The over-expression of each single miRNA affected Blimp-1 expression at different extent, being miR-125b more active than miR-23a in limiting the expression of the α isoform, as compared to cells transfected with unrelated miRNAs and to the empty-vector transfection control. However, the coordinated over-expression of both miRNAs strikingly suppressed the entire expression of Blimp-1α protein (Figure 3A). Next, we assessed the modulation of Blimp-1 in HeLa cells following IFN-α treatment and found that this cytokine induced a clear-cut up-regulation of Blimp-1α protein in a time- and dose-dependent manner, resulting significantly modulated after 48 hours of treatment with the higher dose of cytokine (10,000 U/ml), identical to that used for monocyte-derived DC differentiation (Figure 3B). Remarkably, IFN-α capability to induce Blimp-1α expression was completely lost when HeLa cells were concurrently transfected with miR-23a and miR-125b. Of note, we found that this phenomenon occurred along with a significant induction of Blimp-1αΔ (Figure 3B). Altogether, our data demonstrate that during DC differentiation IFN-α regulates a panel of defined miRNAs, some of which namely miR-23a and miR-125b drive the expression of the active Blimp-1α isoform.

### IFN-α DC and pDC share miRNA-driven control of Blimp-*1* by IFN-α and selected phenotypic, molecular and functional features

**Figure 4:**
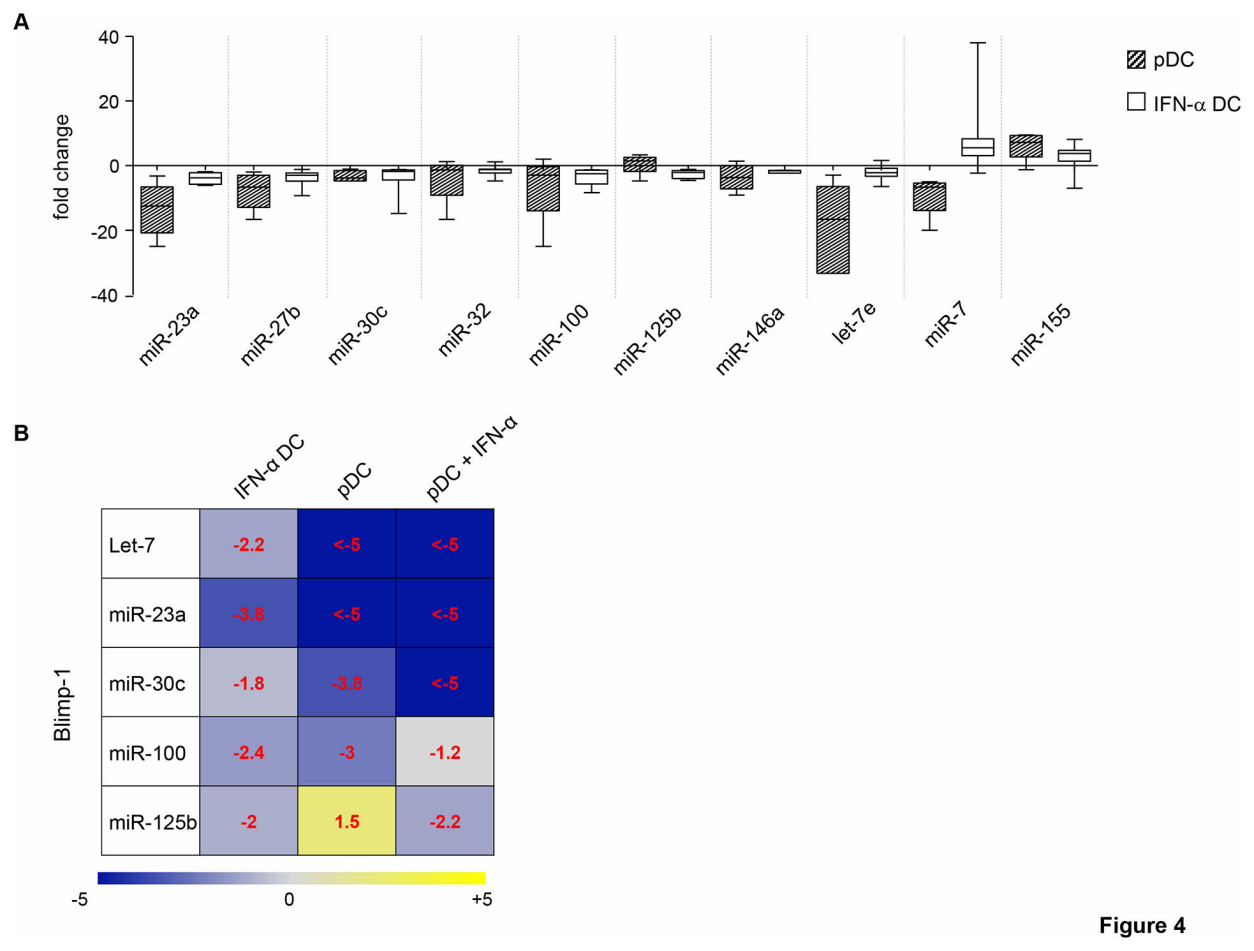
pDC and IFN-α DC share miRNA expression signatures: down-regulation of miR-125b by IFN-α. **A**. Box plot representing expression of 10 miRNAs in peripheral pDC and IFN-α DC analyzed by qRT-PCR. Data represent fold change values of miRNA modulation in pDC and IFN-α DC, with respect to monocytes treated with GM-CSF alone, obtained from 5 and 10 different healthy donors, respectively. **B**. Expression levels of miRNAs targeting Blimp-1 in untreated and IFN-α-treated pDC with respect to IFN-α DC. Median fold-changes are indicated.

**Figure 5:**
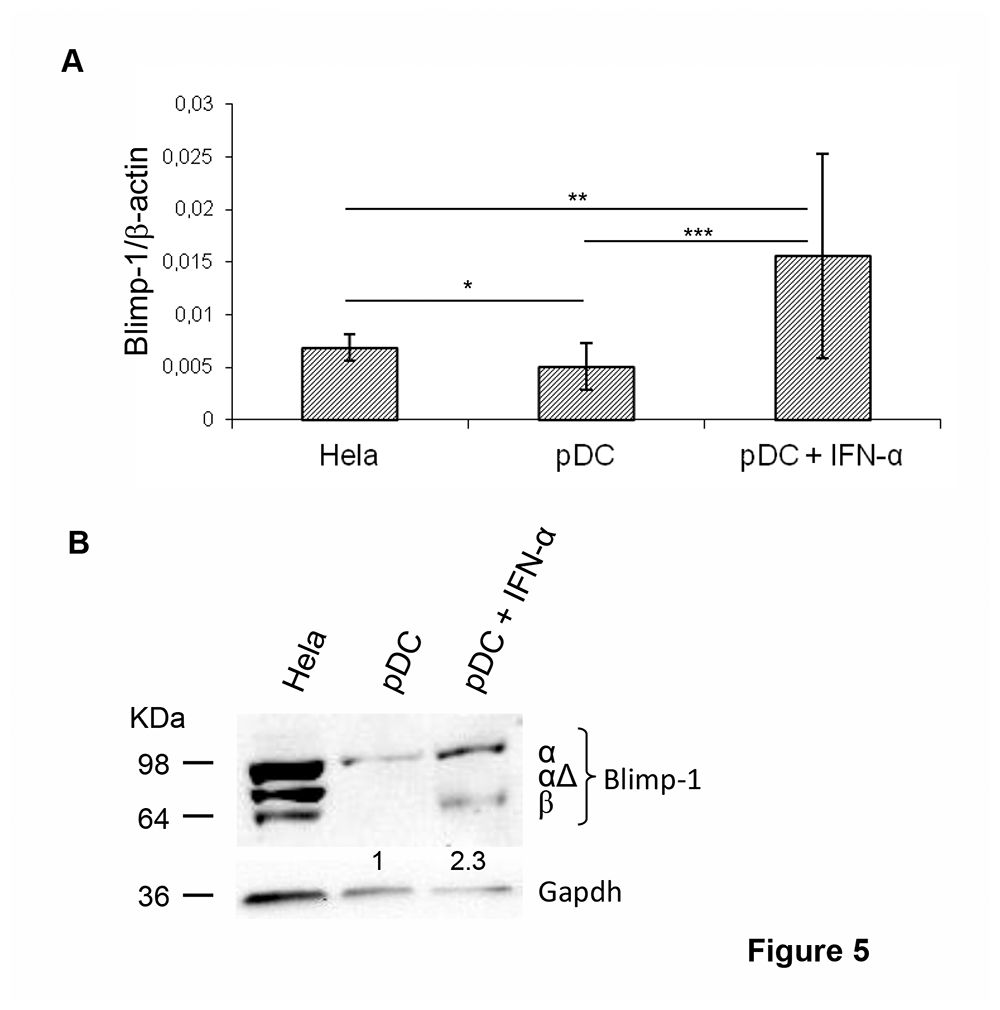
IFN-α induces Blimp-1 expression in peripheral pDC. **A**. Blimp-1 expression in pDC treated or not with IFN-α for 24 hours was analyzed by qRT-PCR. Data are shown as mean ± SD of 3 and 5 independent experiments for untreated and IFN-α-treated pDC respectively. Mann-Whitney test was performed: *p≤0.05, **p≤0.003, ***p=0.0006. **B**. Expression of Blimp-1 isoforms analyzed by western blot in total extracts of pDC and IFN-α-treated pDC. Intensities of bands were measured and values expressed as fold induction with respect to Gapdh, as indicated by numbers. Data from 1 representative experiment out of 3 are shown.

Since IFN-α DC have been reported to display some features similar to those of pDC [37], the expression of 10 miRNAs found tightly regulated in IFN-α DC was assessed in pDC. Interestingly, 8 out of these 10 miRNAs were modulated in the same direction in pDC, being miR-23a, miR-27b, miR-30c, miR-32, miR-100, miR-146a, and let-7e significantly down-modulated and miR-155 up-modulated. Conversely, the expression of miR-7 and miR-125b was found regulated in the opposite direction in the two DC types (Figure 4A). Fold changes and *p*-values of the differentially regulated miRNAs in pDC are shown in Table S4A. We also observed that 4 out of 5 miRNAs shown to regulate Blimp-1 expression were modulated in the same direction in both pDC and IFN-α DC, whereas miR-125b expression was found discordant, being up-modulated in pDC (Figure 4B). Therefore, these two populations share similar miRNA patterns. Next, we investigated whether IFN-α treatment of pDC was able to further modulate the expression pattern of miRNAs and in particular to drive the down-modulation of miR-125b, the only one among those regulating Blimp-1 expression found discordant between pDC and IFN-α DC. The assessment of the expression of miR-23a, miR-30c, miR-100, let-7e and miR-125b in pDC exposed to IFN-α for 24 hours revealed that IFN-α stimulated the down-modulation of miR-125b along with that of miR-30c. Conversely, this cytokine did not further affect the expression of miR-23a as well as let-7e, whereas barely induced miR-100 expression (Figure 4B). Fold change and *p*-value of the differentially regulated miRNAs are shown in Table S4B. Hence, IFN-α treatment of pDC leads essentially to the inhibition of miR-125b expression, which may represent the additional signal needed to modulate the expression of Blimp-1. Lastly, to validate the stimulatory effects of IFN-α on Blimp-1 expression, we assessed the expression of this protein in peripheral pDC cultured or not in the presence of IFN-α for 24 hours. As shown in Figure 5A and B, peripheral pDC expressed basal levels of both Blimp-1 transcripts and of the α isoform protein, whereas IFN-α treatment induced a clear-cut up-modulation of Blimp-1 transcripts and very high levels of Blimp-1α isoform protein. Of interest, IFN-α-treated pDC also showed the expression of low levels of Blimp-1β isoform (Figure 5B). Thus, IFN-α modulates Blimp-1 expression also in peripheral pDC.

**Figure 6:**
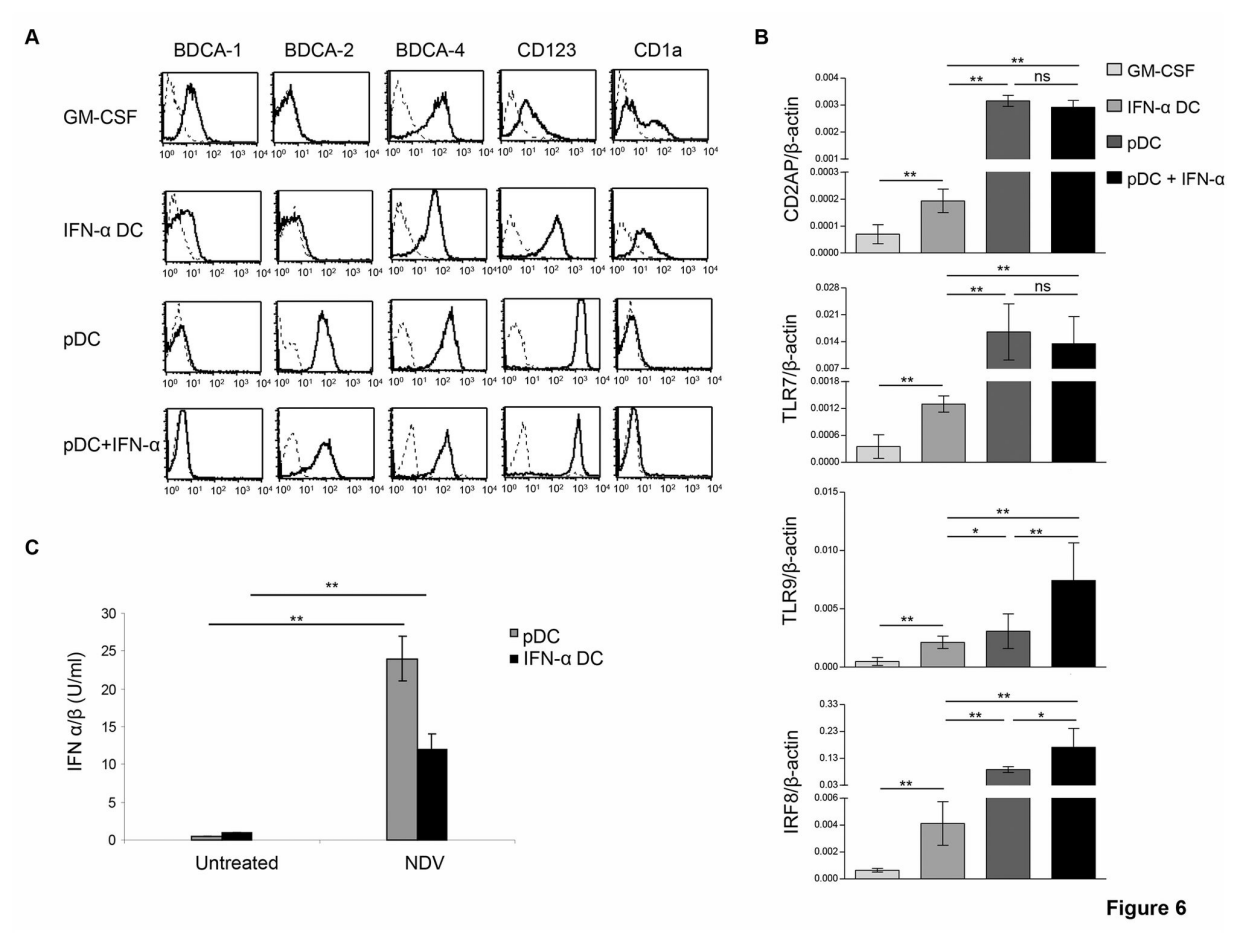
IFN-α DC and peripheral pDC share phenotypic, molecular and functional features. **A**. Flow cytometry analysis of the expression of plasmacytoid or myeloid DC markers in IFN-α DC, untreated or IFN-α-treated pDC and GM-CSF-treated monocytes. Broken line histograms represent isotype controls. Representative data of 1 experiment out of 4 are shown. **B**. Expression of specific pDC molecular markers evaluated by qRT-PCR in the same DC populations indicated in panel **A**. The data are presented as the means ± SD of 3 independent experiments. **C**. Production of IFN-I in IFN-α DC and pDC after in vitro infection with NDV. DC populations were infected with NDV for 1 h. Virus was then washed out and the supernatant was harvested after 18 h incubation and assayed for IFN-I bioactivity, as described in Materials and Methods. Data are representative of 2 independent experiments. Statistical analyses were performed by using Mann-Whitney test, except for IFN-α DC and GM-CSF-treated monocytes comparison for which Wilcoxon test was performed (*p≤0.006, **p≤0.0005, ns = not significant).

To further correlate the effects to IFN-α exposure in monocytes-derived DC and pDC, we characterized these populations for the expression of phenotypic and molecular lineage-associated DC markers. As shown in Figure 6A, IFN-α DC as compared to GM-CSF-treated monocytes exhibited a significant down-modulation of the myeloid marker BDCA-1 and a clear-cut up-regulation of CD123, a marker highly expressed in pDC, even though they were found negative for BDCA-2. Yet, IFN-α DC expressed levels of CD11b and CD11c comparable to those of GM-CSF-treated monocytes (data not shown). Conversely, IL-4 DC showed an expression patter of phenotypic markers perfectly overlaid to that of GM-CSF-treated monocytes (Figure S3A). Next, qRT-PCR analysis revealed that IFN-α DC with respect to GM-CSF-treated monocytes exhibited significant high levels of pDC molecular markers such as Toll-like receptor (TLR)-7, TLR-9, the adaptor protein CD2AP (CD2-associated protein), known to be retained exclusively in normal and neoplastic human pDC [38], and IRF-8, the transcription factor extensively reported to play a crucial role in pDC differentiation and activity [39] (Figure 6B). On the contrary, IL-4 DC expressed all above markers at levels comparable to those of GM-CSF-treated monocytes (Figure S3B) Likewise, the analysis of phenotypic and molecular makers in peripheral pDC exposed for 24 hours to exogenous IFN-α revealed that only TLR9 and IRF-8, and to a lesser extent BDCA-4, were further modulated with respect to untreated pDC (Figure 6A and 6B). Since the major functional feature of pDC is the ability to produce IFN-I upon viral infection, lastly we evaluated whether IFN-α DC exhibited the same functional activity. To this end, we infected pDC and IFN-α DC with NDV and determined IFN-I production in the culture supernatants by biological assay. Interestingly, NDV infection induced significant release of IFN-I in pDC and, although to a lesser extent, in IFN-α DC but it did not stimulate the production of these cytokines in IL-4 DC (Figure 6C and Figure S3C). On the whole, these data suggest that IFN-α DC and pDC share a similar miRNA signature as well as some phenotypic and molecular markers potentially accounting for common functional activities such as the production of IFN-I upon viral infection.

## Discussion

Here we report that IFN-α controls Blimp-1 expression during human DC differentiation associated with down-regulation of a selected pattern of miRNAs among which miR-23a and miR-125b are the major players. These findings imply three novel concepts: i) above and beyond being the master regulator of B-cell differentiation, Blimp-1 is critically implicated in IFN-α-driven differentiation of human DC; ii) IFN-α, other than being the unique effector cytokine of pDC, control the expression of Blimp-1 in pDC as well as in IFN-α DC throughout the modulation of selected miRNAs; iii) IFN-α DC and pDC, over the same Blimp-1-targeting miRNA pattern, share some analogous phenotypic, molecular and functional features ,suggesting the existence of related programs in the two populations.

Recently, IFNs have come into view as strong regulators of miRNAs contributing to antiviral innate immunity [40]. Moreover, a growing body of reports has highlighted the role of miRNAs in the regulation of DC [41,42]. Genes involved in both function and development of DC seem to be affected by the activity of these regulatory molecules [18]. Different expression patterns of miRNAs have been found to regulate pDC *vs* cDC development [22]. Here, we report that DC differentiation driven by IFN-α is characterized by a clear-cut modulation of a specific set of 10 miRNAs of which miR-23a, miR-27b, miR-30, miR-32, miR-100, miR-146a and miR-125b exhibited a significant down-modulation, whereas miR-7 and miR-155 were remarkably up-modulated. Conversely, the *in vitro*-derived IL-4 DC, similarly to cDC, did not exhibit any significant modulation of these as well as other miRNAs across those tested, confirming the potent and specific activity of IFN-α to modulate selected miRNAs during DC functional differentiation. We envisage that the highly conserved miRNA expression pattern of IFN-α DC can be associated with unique functional features displayed by these cells. Increased expression of miR-155 was found to be a general evolutionarily conserved process required for efficient DC maturation and critical for the ability of DC to promote antigen-specific T-cell activation as well as for IFN-I production by human and murine pDC in antiviral innate immunity [43,44]. Accordingly, we found miR-155 significantly over-expressed in IFN-α DC, assigning this up-modulation as a marker of the highly activated phenotype of these cells and providing further support on the positive role of IFN-α in controlling miR-155 expression, which remains one of the miRNAs mostly in use in immune signaling pathways [45]. Among the other IFN-α-driven miRNAs, miR-146a has been reported to negatively regulate DC cross-priming by suppressing IL-12p70 production and its upregulation, following oxLDL stimulation, inhibits the pro-inﬂammatory cytokine release and maturation of DC [46,47]. Hence, the marked decrease of miR-146a in IFN-α DC may suggest an additional mechanism by which IFN-α promotes DC functional properties [5]. Crucially, 5 out of 10 above mentioned miRNAs, at once significantly down-modulated by IFN-α, target Blimp-1, known to be a critical regulator of effector and memory differentiation of B and T lymphocytes [12]. Among the panel of identified Blimp-1 targeting miRNAs, miR-23a and miR-125b have been shown to regulate physiologically hematopoietic development. In fact, abundant expression of both miRNAs is associated to a dramatic decrease in B lymphopoiesis and an increase in myelopoiesis [35,48]. On this basis, we favor the hypothesis that miR-23a and miR-125b, significantly down-modulated by IFN-α during DC differentiation, may cooperatively drive a lineage-skewing towards B cell features via Blimp-1 modulation. This concept is supported by the following findings obtained by using the HeLa cell system: i) concomitant over-expression of miR-23a and miR-125b sharply blocks the expression of the basal levels of Blimp-1α protein isoform; ii) IFN-α stimulates a clear-cut induction of Blimp-1α and this phenomenon is strikingly reversed by co-expression of miR-23a and miR-125b. Of interest, although in HeLa cells the concomitant exposure to IFN-α and miR-23a/miR-125b over-expression associated with full loss of Blimp-1α isoform but enhanced levels of Blimp-1αΔ, any modulation of this latter isoform protein was not observed in IFN-α DC as well as in IFN-α-treated pDC. Recently, it has been reported that Blimp-1 protein levels increase dramatically during DC differentiation and the full-length Blimp-1 protein is strongly expressed by LPS-treated bone marrow-derived DC [49]. In line with these findings, our result supports the concept that IFN-α exposure leads to partially mature monocytes-derived DC as well as to activation of peripheral pDC. Overall, our data indicate that during the process of DC differentiation from human CD14+ monocytes IFN-α modulates the expression of miR-23a and miR-125b thus controlling Blimp-1 expression in a time-dependent manner.

pDC secrete large amounts of IFN-I in response to viruses and promote immunity [8]. These cells also exhibit large amounts of MHC class I (MHC-I) molecules stored in endosomes and, upon signals of maturation, may become competent antigen-presenting cells and efficiently induce CD8+ T cell responses [10]. Similarly, IFN-α DC display a strong ability to rearrange MHC-I molecules associated to a remarkable cross-priming of CD8+ T cell [5]. Here, we report that IFN-α DC, similarly to pDC, are able to produce a consistent amount of IFN-I upon NDV infection. Moreover, IFN-α-driven differentiation of monocytes-derived DC leads to acquire several lineage-pDC surface and molecular markers, such as CD123, CD2AP, TLR7, TLR9 and IRF-8 [38]. Moreover, IFN-α DC and pDC were found to share a similar expression pattern of miRNAs associated to a significant expression of Blimp-1. Of interest, following exposure with exogenous IFN-α, pDC further modulated miR-23a and miR-125b and increased Blimp-1 expression. Altogether, these findings suggest that IFN-α exposure both during human monocyte-derived DC differentiation, as it occurs in IFN-α DC at 3 days of culture, and in the activation stage of pDC can lead to the acquisition of shared functional competences. Therefore, we foresee that *in vivo*, when IFN-α is released in the course of pathological conditions in human, a sequential control of Blimp-1 expression can occur potentially impacting the functional competence of both pDC and other naturally occurring DC rapidly generated from monocytes in response to danger signals.

Blimp-1 has been reported to regulate negatively mouse CD11c^high^CD8α^-^ cDC development, whereas its expression is increased during cDC maturation mediated by NF-κB and p38 MAPK pathways [15]. Likewise, it has been reported that reconstitution of Blimp-1 in deficient DC reverses inflammatory phenotype [50]. Moreover, the fine control of Blimp-1 expression in mouse DC has been shown to avoid an aberrant activation of the adaptive immune system, leading to the development of a lupus-like serology in a gender-specific manner [51]. All these findings reflect the capability of Blimp-1 to act within the immune system during development stages of diverse immune populations controlling numerous gene-expression programs [52], and here we report additional data supporting this function of Blimp-1 also in pDC as well in IFN-driven monocytes-derived DC. It is noteworthy that in IFN-α DC the induction of Blimp-1 is time-dependent reaching the peak at day 3 of differentiation, when the cells are fully differentiated [32], then declining to undetectable levels the day after. We envisage that the control of Blimp-1 via IFN-α/miRNAs in a time-dependent manner, both in IFN-α DC and in pDC, may represent a fine mechanism to tightly regulate DC full maturation and function and simultaneously to ensure a proper homeostasis of the immune system when it is needed. For instance, Blimp-1 may negatively control the production of effector cytokines, such TNF-α or IL-6, skewing DC to tolerance rather than immunity once host immune homeostasis requires to be restored [33,51]. Likewise, since Blimp-1 negatively controls CIITA-dependent MHC-II expression as well as MHC-I levels in response to IFN-γ [16,53], it may direct a fine mechanism behind the different antigen presentation potential characterizing pDC at different developmental stages [54,55]. In this context, it has been reported that the induction of Blimp-1 during DC maturation abrogates activation of CIITApI mediated by IRF-8, the well-known transcription factor implicated in pDC differentiation [28], thus establishing a coordinate mechanism to control MHC-II expression in these cells [16]. Since these events may occur during an active immune response, when pDC need to be fully maturated and activated first, then to sense and respond to danger signals and finally to shut down their functional activities [56], we favor the hypothesis that IFN-α-induced Blimp-1 and its fine-tunes functional capability play an important regulatory role in pDC-associated features. Hence, although the fine effects of Blimp-1 expression in pDC remain to be addressed, here we provide evidence for a potential role of Blimp-1 in determining the functional identity of this DC subset and envisage that this phenomenon maybe under the control of IFN-α mainly via regulation of miR-23a and miR-125b. We also demonstrate that IFN-α DC and pDC share some common phenotypic, molecular and functional properties. These findings renovate the interest for IFN-α DC as a potent and suitable therapeutic tool to be exploited in parallel with pDC for inducing favorable immunity in the treatment of some human diseases, including cancer and some chronic viral infections.

## Supporting Information

Table S1 **Fold change values for 30 selected miRNAs in IFN-α** **DC and IL-4 DC vs**. **GM-CSF-treated monocytes**.
(PPTX)

Table S2 **miRNAs differentially modulated by IFN-α and IL-4 during DC differentiation.** 9 out of 10 miRNAs and 3 out of 9 miRNAs resulted to be significantly modulated respectively in IFN-α DC and IL-4 DC.
(PPTX)

Table S3 **Genes targeted by miRNAs in IFN-α DC.** Genes predicted to be targeted by 5 to 7 miRNA found to be modulated in IFN-α DC, by means of miRGator program.
(PPTX)

Table S4 **miRNA signature of pDC and IFN-α-treated pDC.** A. Median fold-change of IFN-α-related miRNAs in pDC; B. Median fold-change of 5 miRNAs predicted to target the *PRDM-1*/Blimp1 gene, upon IFN-α treatment of pDC.
(PPTX)

Figure S1 **Blimp-1, miR23a and miR125b expression in TNF-a-activated IL-4** dC.A. Blimp-1 expression was analyzed by qRT-PCR in the indicated DC populations generated as reported in Materials and Methods. Data are expressed as mean ± SD of 3 independent experiments. Mann-Whitney test was performed: *p=0.002, **p≤0.0001. **B**. MiR-23a and miR-125b quantification was carried out by qRT-PCR as reported in Material and Methods and fold changes of miRNA expression in the indicated DC populations obtained by 2 different donors (Don. A and Don. B) were calculated using GM-CSF-treated monocytes as control.
(PPT)

Figure S2 **miR-23a and miR-125b expression in transfected HeLa cells.** HeLa cells were transfected with indicated miRNAs whose over-expression was analyzed by northern blot. Control represent Hela with empty plasmid. Small nuclear U6 (snU6) was used as internal control. 1 representative experiment out of 3 is shown. Intensities of miR-23a and miR-125b bands, alone or in combination, were measured and expressed as arbitrary units on the right of the panel. Values are normalized to snU6 and expressed as mean ± SD of 3 independent experiments.
(PPT)

Figure S3 **Phenotypic, molecular and functional features of IL-4** dC.A. Flow cytometry analysis of lineage-DC markers in IL-4 DC and GM-CSF-treated monocytes. Broken line histograms represent isotype controls. Representative data of 1 experiment out of 3 are shown. **B**. Expression of pDC–related molecular markers evaluated by qRT-PCR in the same DC populations indicated in panel A. The data are presented as the means ± SD of 3 independent experiments. **C**. Production of IFN-I in IL-4 DC and pDC after in vitro infection with NDV. DC populations were infected with NDV for 1 hour. Virus was then washed out and the supernatant was harvested after 18 hour incubation and assayed for IFN-I bioactivity, as described in Materials and Methods. Data are representative of 2 independent experiments. Statistical analyses were performed by using Mann-Whitney test (*p≤0.0001, ns = not significant).
(PPT)
