# Supplementary material for: IFN-α Regulates Blimp-1 Expression via miR-23a and miR-125b in Both Monocytes-Derived DC and pDC
Source: PLoS One. 2013 Aug 16;8(8):e72833. doi: 10.1371/journal.pone.0072833 (PMC3745402; doi:10.1371/journal.pone.0072833)
Supplement: Table S1 — Fold change values for 30 selected miRNAs in IFN-α DC and IL-4 DC vs. GM-CSF-treated monocytes. (PPTX) [file pone.0072833.s001.pptx]

## Slide 1
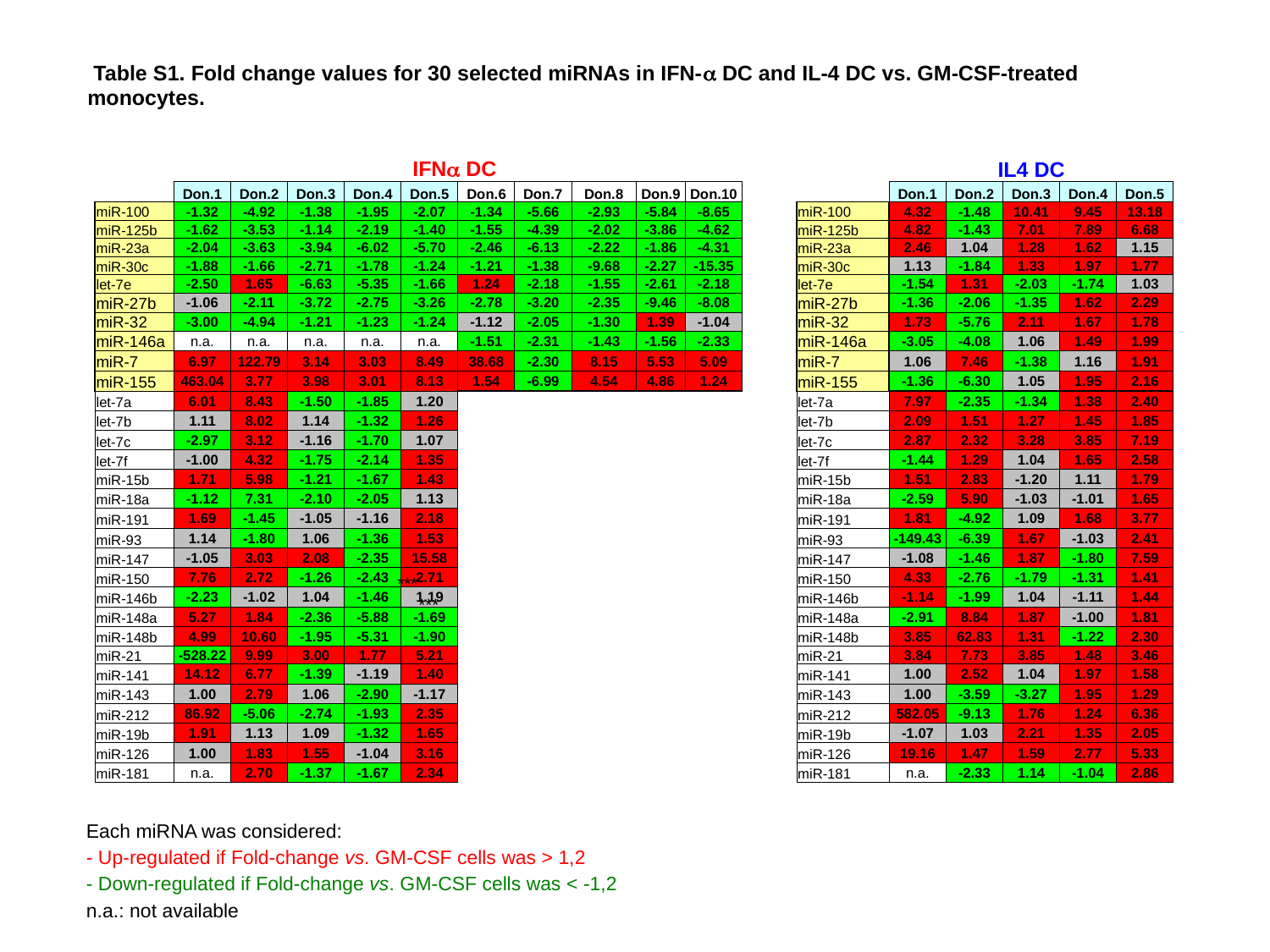

Table S1. Fold change values for 30 selected miRNAs in IFN-α DC and IL-4 DC vs. GM-CSF-treated monocytes.
| | IFNα DC | | | | | | | | | | | | IL4 DC | | | | |
| --- | --- | --- | --- | --- | --- | --- | --- | --- | --- | --- | --- | --- | --- | --- | --- | --- | --- |
| | Don.1 | Don.2 | Don.3 | Don.4 | Don.5 | Don.6 | Don.7 | Don.8 | Don.9 | Don.10 | | | Don.1 | Don.2 | Don.3 | Don.4 | Don.5 |
| miR-100 | -1.32 | -4.92 | -1.38 | -1.95 | -2.07 | -1.34 | -5.66 | -2.93 | -5.84 | -8.65 | | miR-100 | 4.32 | -1.48 | 10.41 | 9.45 | 13.18 |
| miR-125b | -1.62 | -3.53 | -1.14 | -2.19 | -1.40 | -1.55 | -4.39 | -2.02 | -3.86 | -4.62 | | miR-125b | 4.82 | -1.43 | 7.01 | 7.89 | 6.68 |
| miR-23a | -2.04 | -3.63 | -3.94 | -6.02 | -5.70 | -2.46 | -6.13 | -2.22 | -1.86 | -4.31 | | miR-23a | 2.46 | 1.04 | 1.28 | 1.62 | 1.15 |
| miR-30c | -1.88 | -1.66 | -2.71 | -1.78 | -1.24 | -1.21 | -1.38 | -9.68 | -2.27 | -15.35 | | miR-30c | 1.13 | -1.84 | 1.33 | 1.97 | 1.77 |
| let-7e | -2.50 | 1.65 | -6.63 | -5.35 | -1.66 | 1.24 | -2.18 | -1.55 | -2.61 | -2.18 | | let-7e | -1.54 | 1.31 | -2.03 | -1.74 | 1.03 |
| miR-27b | -1.06 | -2.11 | -3.72 | -2.75 | -3.26 | -2.78 | -3.20 | -2.35 | -9.46 | -8.08 | | miR-27b | -1.36 | -2.06 | -1.35 | 1.62 | 2.29 |
| miR-32 | -3.00 | -4.94 | -1.21 | -1.23 | -1.24 | -1.12 | -2.05 | -1.30 | 1.39 | -1.04 | | miR-32 | 1.73 | -5.76 | 2.11 | 1.67 | 1.78 |
| miR-146a | n.a. | n.a. | n.a. | n.a. | n.a. | -1.51 | -2.31 | -1.43 | -1.56 | -2.33 | | miR-146a | -3.05 | -4.08 | 1.06 | 1.49 | 1.99 |
| miR-7 | 6.97 | 122.79 | 3.14 | 3.03 | 8.49 | 38.68 | -2.30 | 8.15 | 5.53 | 5.09 | | miR-7 | 1.06 | 7.46 | -1.38 | 1.16 | 1.91 |
| miR-155 | 463.04 | 3.77 | 3.98 | 3.01 | 8.13 | 1.54 | -6.99 | 4.54 | 4.86 | 1.24 | | miR-155 | -1.36 | -6.30 | 1.05 | 1.95 | 2.16 |
| let-7a | 6.01 | 8.43 | -1.50 | -1.85 | 1.20 | | | | | | | let-7a | 7.97 | -2.35 | -1.34 | 1.38 | 2.40 |
| let-7b | 1.11 | 8.02 | 1.14 | -1.32 | 1.26 | | | | | | | let-7b | 2.09 | 1.51 | 1.27 | 1.45 | 1.85 |
| let-7c | -2.97 | 3.12 | -1.16 | -1.70 | 1.07 | | | | | | | let-7c | 2.87 | 2.32 | 3.28 | 3.85 | 7.19 |
| let-7f | -1.00 | 4.32 | -1.75 | -2.14 | 1.35 | | | | | | | let-7f | -1.44 | 1.29 | 1.04 | 1.65 | 2.58 |
| miR-15b | 1.71 | 5.98 | -1.21 | -1.67 | 1.43 | | | | | | | miR-15b | 1.51 | 2.83 | -1.20 | 1.11 | 1.79 |
| miR-18a | -1.12 | 7.31 | -2.10 | -2.05 | 1.13 | | | | | | | miR-18a | -2.59 | 5.90 | -1.03 | -1.01 | 1.65 |
| miR-191 | 1.69 | -1.45 | -1.05 | -1.16 | 2.18 | | | | | | | miR-191 | 1.81 | -4.92 | 1.09 | 1.68 | 3.77 |
| miR-93 | 1.14 | -1.80 | 1.06 | -1.36 | 1.53 | | | | | | | miR-93 | -149.43 | -6.39 | 1.67 | -1.03 | 2.41 |
| miR-147 | -1.05 | 3.03 | 2.08 | -2.35 | 15.58 | | | | | | | miR-147 | -1.08 | -1.46 | 1.87 | -1.80 | 7.59 |
| miR-150 | 7.76 | 2.72 | -1.26 | -2.43 | 2.71 | | | | | | | miR-150 | 4.33 | -2.76 | -1.79 | -1.31 | 1.41 |
| miR-146b | -2.23 | -1.02 | 1.04 | -1.46 | 1.19 | | | | | | | miR-146b | -1.14 | -1.99 | 1.04 | -1.11 | 1.44 |
| miR-148a | 5.27 | 1.84 | -2.36 | -5.88 | -1.69 | | | | | | | miR-148a | -2.91 | 8.84 | 1.87 | -1.00 | 1.81 |
| miR-148b | 4.99 | 10.60 | -1.95 | -5.31 | -1.90 | | | | | | | miR-148b | 3.85 | 62.83 | 1.31 | -1.22 | 2.30 |
| miR-21 | -528.22 | 9.99 | 3.00 | 1.77 | 5.21 | | | | | | | miR-21 | 3.84 | 7.73 | 3.85 | 1.48 | 3.46 |
| miR-141 | 14.12 | 6.77 | -1.39 | -1.19 | 1.40 | | | | | | | miR-141 | 1.00 | 2.52 | 1.04 | 1.97 | 1.58 |
| miR-143 | 1.00 | 2.79 | 1.06 | -2.90 | -1.17 | | | | | | | miR-143 | 1.00 | -3.59 | -3.27 | 1.95 | 1.29 |
| miR-212 | 86.92 | -5.06 | -2.74 | -1.93 | 2.35 | | | | | | | miR-212 | 582.05 | -9.13 | 1.76 | 1.24 | 6.36 |
| miR-19b | 1.91 | 1.13 | 1.09 | -1.32 | 1.65 | | | | | | | miR-19b | -1.07 | 1.03 | 2.21 | 1.35 | 2.05 |
| miR-126 | 1.00 | 1.83 | 1.55 | -1.04 | 3.16 | | | | | | | miR-126 | 19.16 | 1.47 | 1.59 | 2.77 | 5.33 |
| miR-181 | n.a. | 2.70 | -1.37 | -1.67 | 2.34 | | | | | | | miR-181 | n.a. | -2.33 | 1.14 | -1.04 | 2.86 |
***
***
| Each miRNA was considered: |
| --- |
| - Up-regulated if Fold-change vs. GM-CSF cells was > 1,2 |
| - Down-regulated if Fold-change vs. GM-CSF cells was < -1,2 |
| n.a.: not available |
