# Supplementary material for: IFN-α Regulates Blimp-1 Expression via miR-23a and miR-125b in Both Monocytes-Derived DC and pDC
Source: PLoS One. 2013 Aug 16;8(8):e72833. doi: 10.1371/journal.pone.0072833 (PMC3745402; doi:10.1371/journal.pone.0072833)
Supplement: Table S2 — miRNAs differentially modulated by IFN-α and IL-4 during DC differentiation. 9 out of 10 miRNAs and 3 out of 9 miRNAs resulted to be significantly modulated respectively in IFN-α DC and IL-4 DC. (PPTX) [file pone.0072833.s002.pptx]

## Slide 1
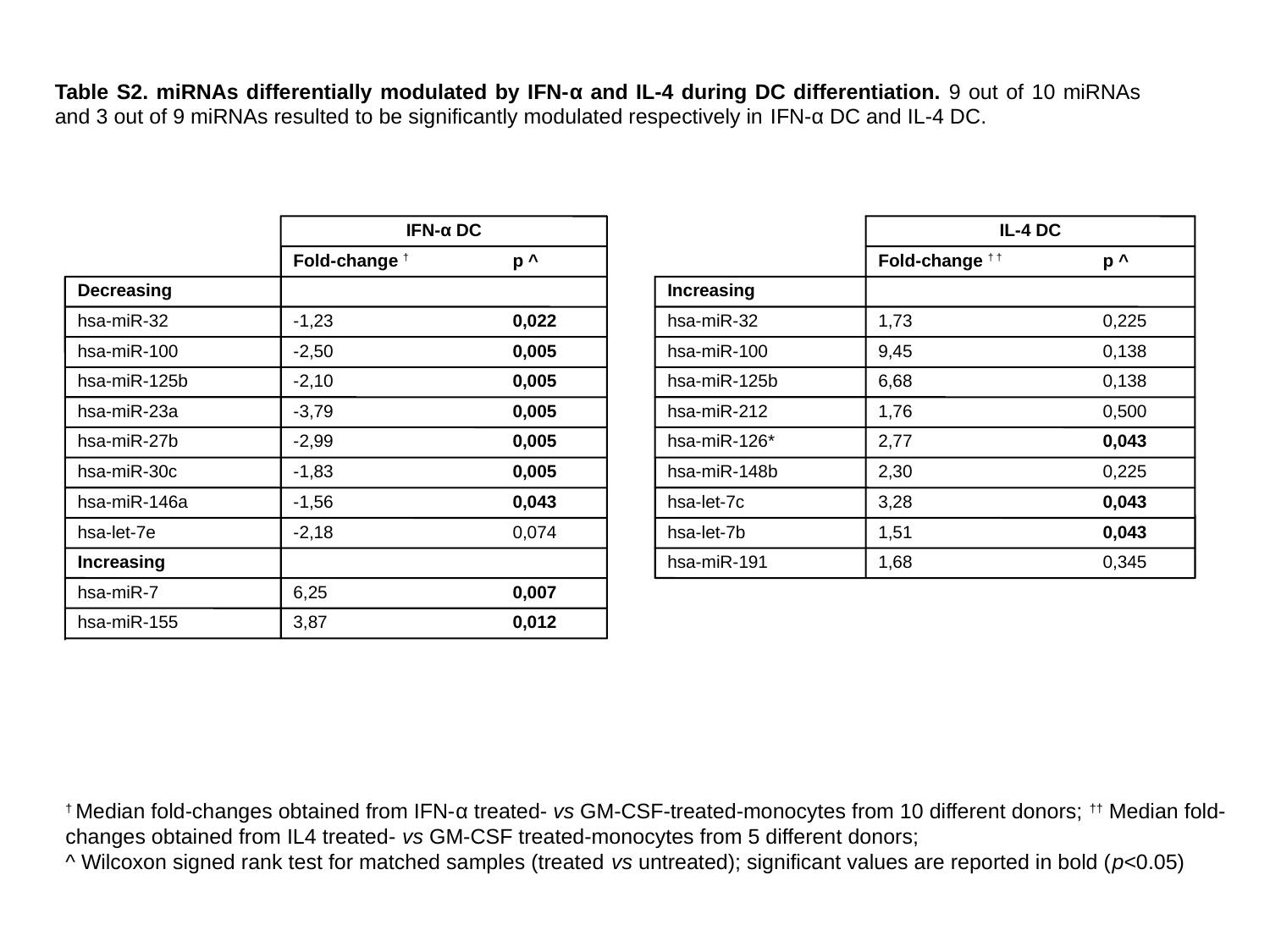

Table S2. miRNAs differentially modulated by IFN-α and IL-4 during DC differentiation. 9 out of 10 miRNAs and 3 out of 9 miRNAs resulted to be significantly modulated respectively in IFN-α DC and IL-4 DC.
IFN-α DC
Fold-change †
p ^
Decreasing
hsa-miR-32
-1,23
0,022
hsa-miR-100
-2,50
0,005
hsa-miR-125b
-2,10
0,005
hsa-miR-23a
-3,79
0,005
hsa-miR-27b
-2,99
0,005
hsa-miR-30c
-1,83
0,005
hsa-miR-146a
-1,56
0,043
hsa-let-7e
-2,18
0,074
Increasing
hsa-miR-7
6,25
0,007
hsa-miR-155
3,87
0,012
IL-4 DC
Fold-change † †
p ^
Increasing
hsa-miR-32
1,73
0,225
hsa-miR-100
9,45
0,138
hsa-miR-125b
6,68
0,138
hsa-miR-212
1,76
0,500
hsa-miR-126*
2,77
0,043
hsa-miR-148b
2,30
0,225
hsa-let-7c
3,28
0,043
hsa-let-7b
1,51
0,043
hsa-miR-191
1,68
0,345
† Median fold-changes obtained from IFN-α treated- vs GM-CSF-treated-monocytes from 10 different donors; †† Median fold-changes obtained from IL4 treated- vs GM-CSF treated-monocytes from 5 different donors;
^ Wilcoxon signed rank test for matched samples (treated vs untreated); significant values are reported in bold (p<0.05)
