# Supplementary material for: IFN-α Regulates Blimp-1 Expression via miR-23a and miR-125b in Both Monocytes-Derived DC and pDC
Source: PLoS One. 2013 Aug 16;8(8):e72833. doi: 10.1371/journal.pone.0072833 (PMC3745402; doi:10.1371/journal.pone.0072833)
Supplement: Table S3 — Genes targeted by miRNAs in IFN-α DC. Genes predicted to be targeted by 5 to 7 miRNA found to be modulated in IFN-α DC, by means of miRGator program. (PPTX) [file pone.0072833.s003.pptx]

## Slide 1
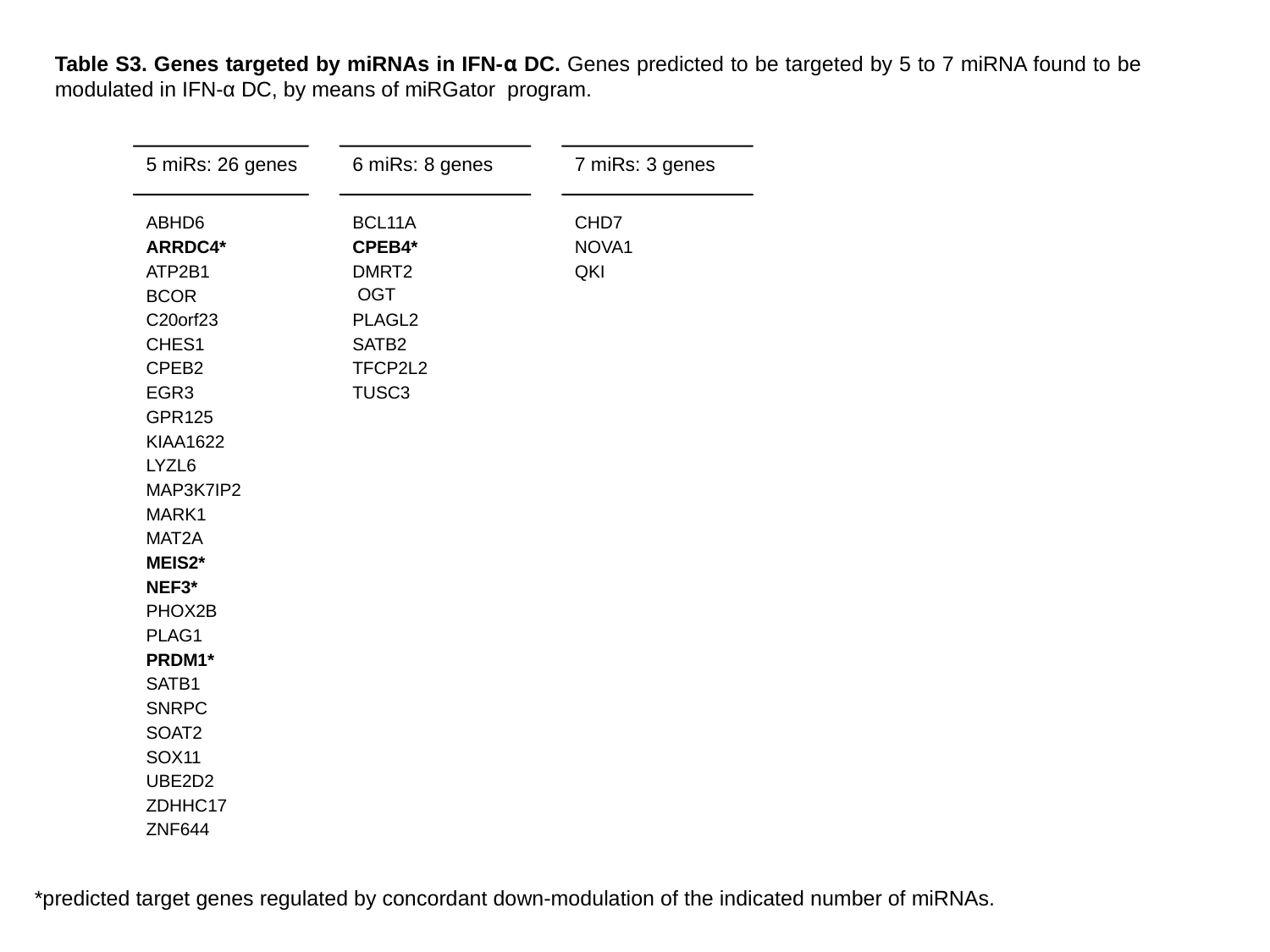

Table S3. Genes targeted by miRNAs in IFN-α DC. Genes predicted to be targeted by 5 to 7 miRNA found to be modulated in IFN-α DC, by means of miRGator program.
5 miRs: 26 genes
6 miRs: 8 genes
7 miRs: 3 genes
ABHD6
BCL11A
CHD7
ARRDC4*
CPEB4*
NOVA1
ATP2B1
DMRT2
QKI
OGT
BCOR
C20orf23
PLAGL2
CHES1
SATB2
CPEB2
TFCP2L2
EGR3
TUSC3
GPR125
KIAA1622
LYZL6
MAP3K7IP2
MARK1
MAT2A
MEIS2*
NEF3*
PHOX2B
PLAG1
PRDM1*
SATB1
SNRPC
SOAT2
SOX11
UBE2D2
ZDHHC17
ZNF644
*predicted target genes regulated by concordant down-modulation of the indicated number of miRNAs.
