# Supplementary material for: IFN-α Regulates Blimp-1 Expression via miR-23a and miR-125b in Both Monocytes-Derived DC and pDC
Source: PLoS One. 2013 Aug 16;8(8):e72833. doi: 10.1371/journal.pone.0072833 (PMC3745402; doi:10.1371/journal.pone.0072833)
Supplement: Table S4 — miRNA signature of pDC and IFN-α-treated pDC. A. Median fold-change of IFN-α-related miRNAs in pDC; B. Median fold-change of 5 miRNAs predicted to target the PRDM-1/Blimp1 gene, upon IFN-α treatment of pDC. (PPTX) [file pone.0072833.s004.pptx]

## Slide 1
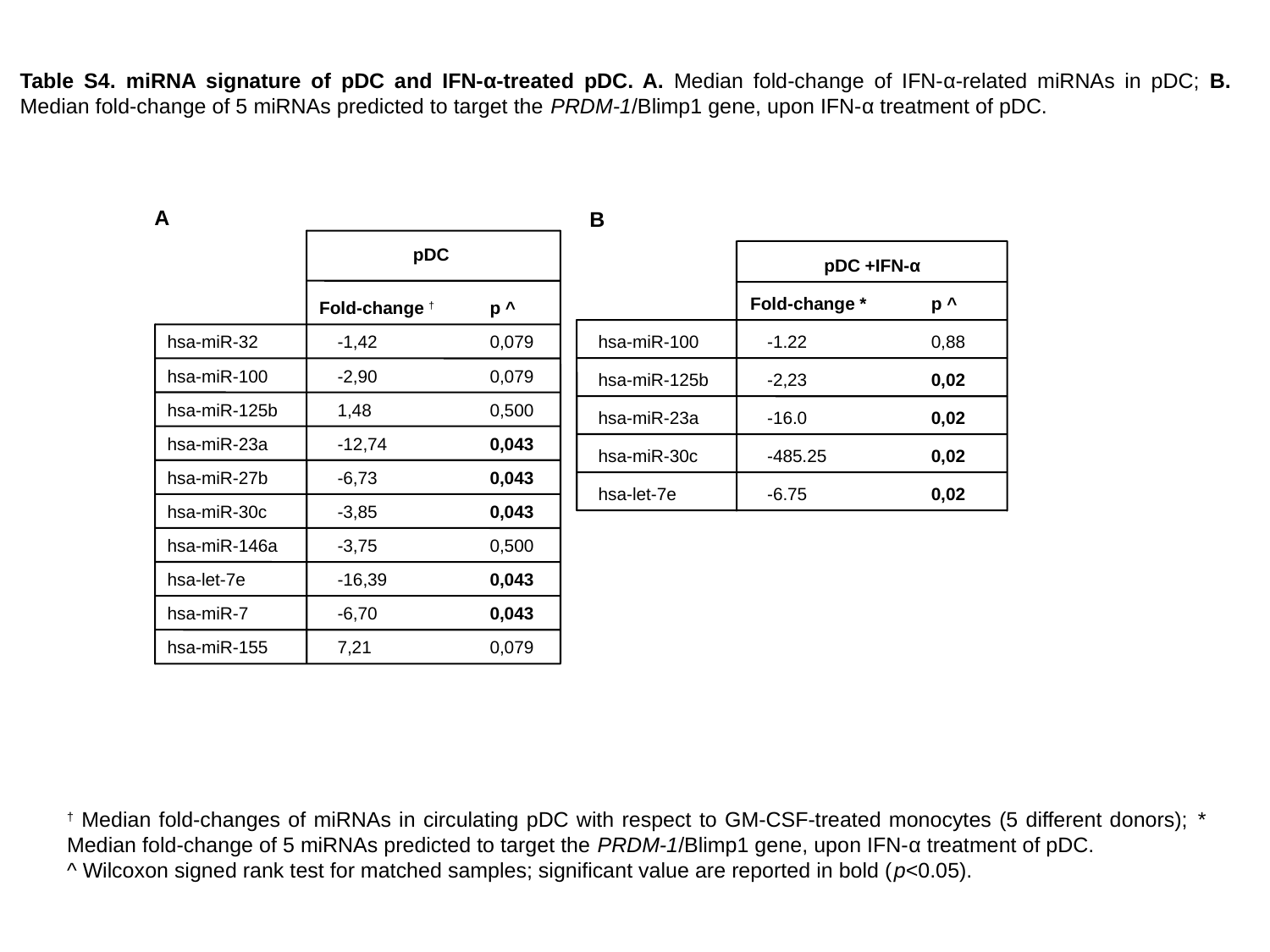

Table S4. miRNA signature of pDC and IFN-α-treated pDC. A. Median fold-change of IFN-α-related miRNAs in pDC; B. Median fold-change of 5 miRNAs predicted to target the PRDM-1/Blimp1 gene, upon IFN-α treatment of pDC.
A
B
pDC
Fold-change †
p ^
hsa-miR-32
-1,42
0,079
hsa-miR-100
-2,90
0,079
hsa-miR-125b
1,48
0,500
hsa-miR-23a
-12,74
0,043
hsa-miR-27b
-6,73
0,043
hsa-miR-30c
-3,85
0,043
hsa-miR-146a
-3,75
0,500
hsa-let-7e
-16,39
0,043
hsa-miR-7
-6,70
0,043
hsa-miR-155
7,21
0,079
pDC +IFN-α
Fold-change *
p ^
hsa-miR-100
-1.22
0,88
hsa-miR-125b
-2,23
0,02
hsa-miR-23a
-16.0
0,02
hsa-miR-30c
-485.25
0,02
hsa-let-7e
-6.75
0,02
† Median fold-changes of miRNAs in circulating pDC with respect to GM-CSF-treated monocytes (5 different donors); * Median fold-change of 5 miRNAs predicted to target the PRDM-1/Blimp1 gene, upon IFN-α treatment of pDC.
^ Wilcoxon signed rank test for matched samples; significant value are reported in bold (p<0.05).
