# Supplementary material for: IFN-α Regulates Blimp-1 Expression via miR-23a and miR-125b in Both Monocytes-Derived DC and pDC
Source: PLoS One. 2013 Aug 16;8(8):e72833. doi: 10.1371/journal.pone.0072833 (PMC3745402; doi:10.1371/journal.pone.0072833)
Supplement: Figure S1 — Blimp-1, miR23a and miR125b expression in TNF-a-activated IL-4 dC.A. Blimp-1 expression was analyzed by qRT-PCR in the indicated DC populations generated as reported in Materials and Methods. Data are expressed as mean ± SD of 3 independent experiments. Mann-Whitney test was performed: *p=0.002, **p≤0.0001. B. MiR-23a and miR-125b quantification was carried out by qRT-PCR as reported in Material and Methods and fold changes of miRNA expression in the indicated DC populations obtained by 2 different donors (Don. A and Don. B) were calculated using GM-CSF-treated monocytes as control. (PPT) [file pone.0072833.s005.ppt]

## Slide 1
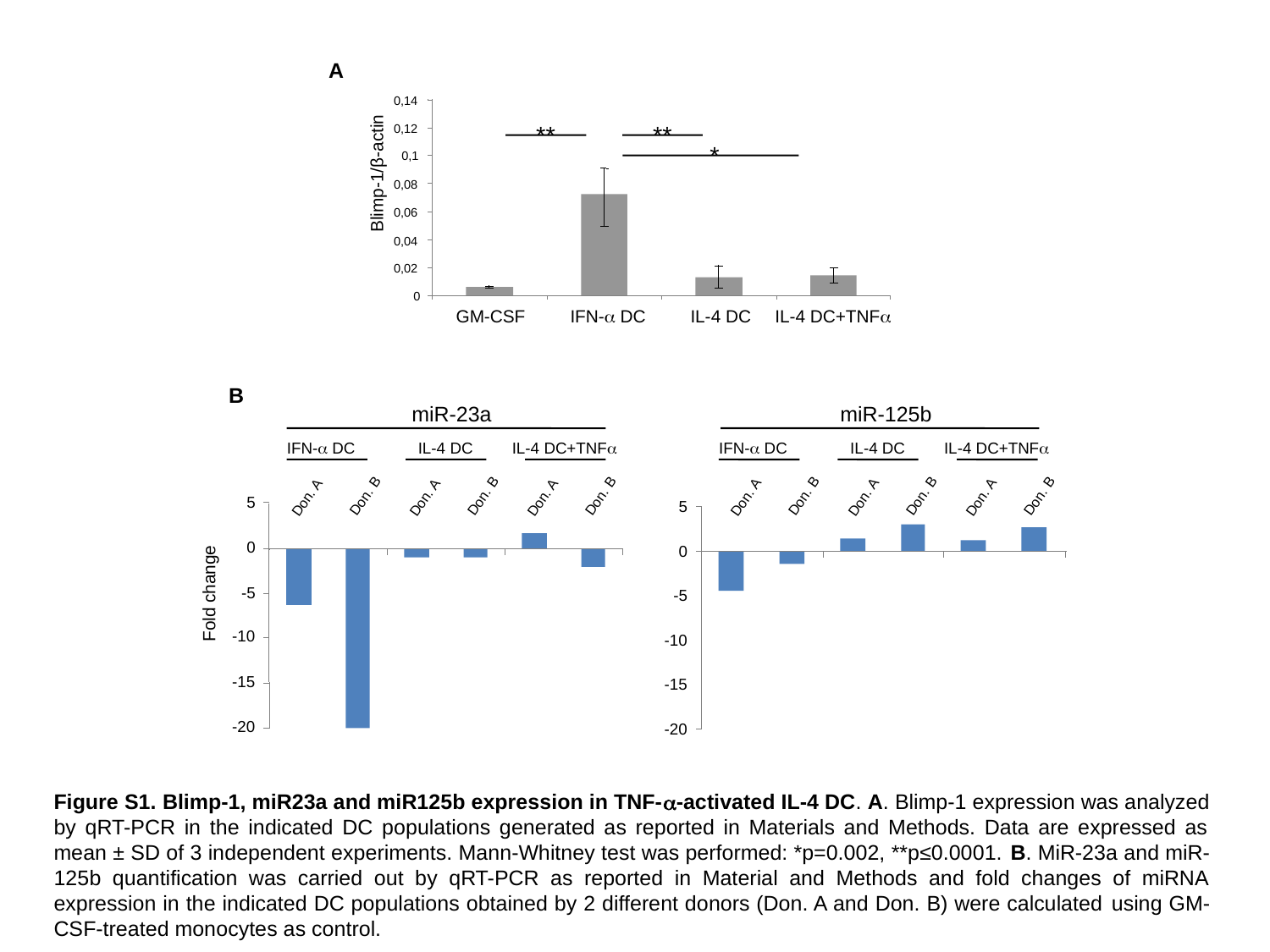

A
0,14
**
**
0,12
*
0,1
Blimp-1/β-actin
0,08
0,06
0,04
0,02
0
GM-CSF
IFN-α DC
IL-4 DC
IL-4 DC+TNFα
B
miR-23a
IFN-α DC
IL-4 DC
IL-4 DC+TNFα
Don. A
Don. B
Don. A
Don. B
Don. A
Don. B
5
0
-5
-10
-15
-20
miR-125b
IFN-α DC
IL-4 DC
IL-4 DC+TNFα
Don. A
Don. A
Don. A
Don. B
Don. B
Don. B
5
0
Fold change
-5
-10
-15
-20
Figure S1. Blimp-1, miR23a and miR125b expression in TNF-α-activated IL-4 DC. A. Blimp-1 expression was analyzed by qRT-PCR in the indicated DC populations generated as reported in Materials and Methods. Data are expressed as mean ± SD of 3 independent experiments. Mann-Whitney test was performed: *p=0.002, **p≤0.0001. B. MiR-23a and miR-125b quantification was carried out by qRT-PCR as reported in Material and Methods and fold changes of miRNA expression in the indicated DC populations obtained by 2 different donors (Don. A and Don. B) were calculated using GM-CSF-treated monocytes as control.
