# Supplementary material for: IFN-α Regulates Blimp-1 Expression via miR-23a and miR-125b in Both Monocytes-Derived DC and pDC
Source: PLoS One. 2013 Aug 16;8(8):e72833. doi: 10.1371/journal.pone.0072833 (PMC3745402; doi:10.1371/journal.pone.0072833)
Supplement: Figure S2 — miR-23a and miR-125b expression in transfected HeLa cells. HeLa cells were transfected with indicated miRNAs whose over-expression was analyzed by northern blot. Control represent Hela with empty plasmid. Small nuclear U6 (snU6) was used as internal control. 1 representative experiment out of 3 is shown. Intensities of miR-23a and miR-125b bands, alone or in combination, were measured and expressed as arbitrary units on the right of the panel. Values are normalized to snU6 and expressed as mean ± SD of 3 independent experiments. (PPT) [file pone.0072833.s006.ppt]

## Slide 1
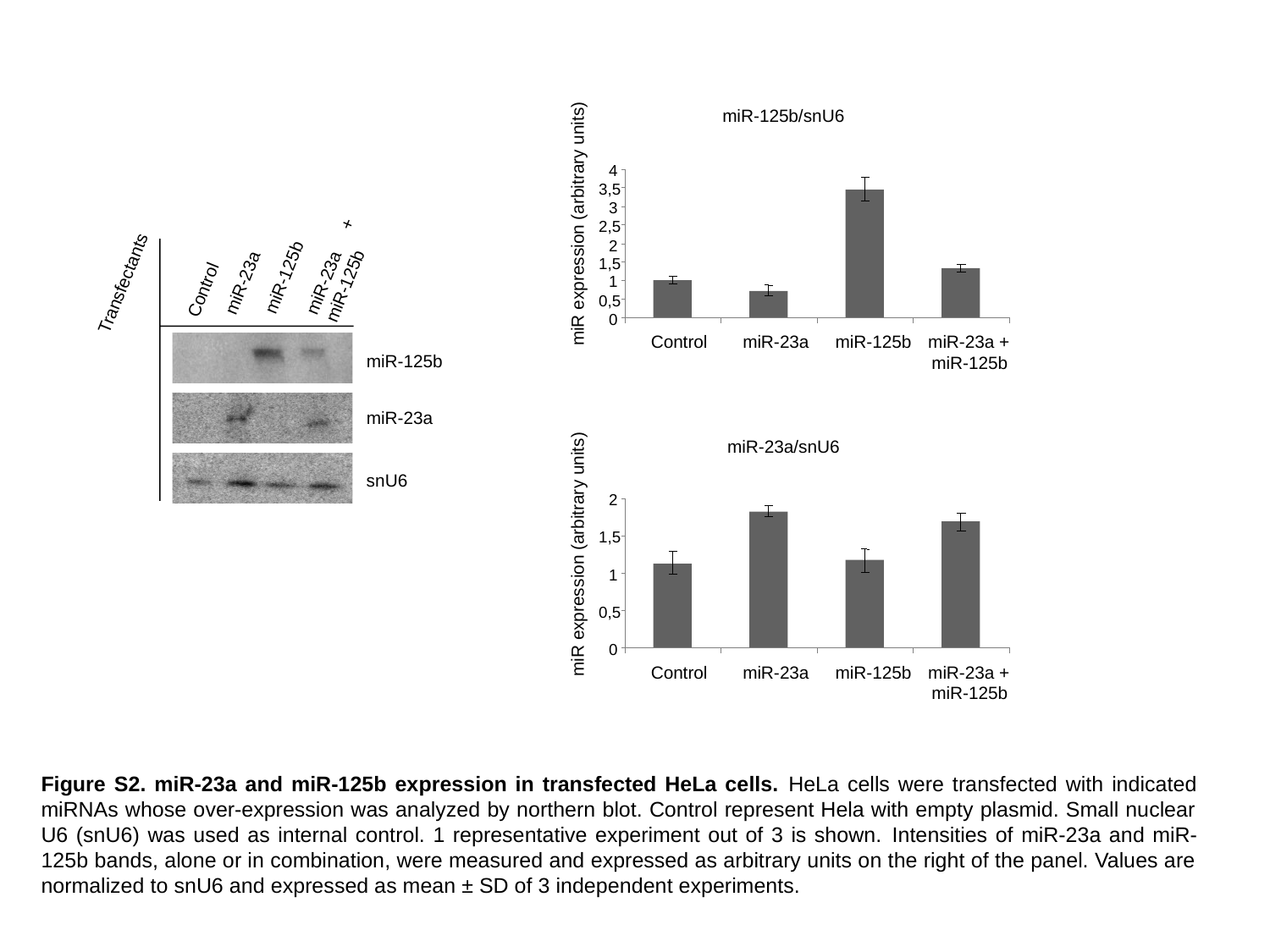

miR-125b/snU6
4
3,5
3
miR-23a + miR-125b
miR-125b
miR-23a
Control
miR-125b
miR-23a
snU6
miR expression (arbitrary units)
2,5
2
1,5
Transfectants
1
0,5
0
Control
miR-23a
miR-125b
miR-23a +
miR-125b
miR-23a/snU6
2
1,5
miR expression (arbitrary units)
1
0,5
0
Control
miR-23a
miR-125b
miR-23a +
miR-125b
Figure S2. miR-23a and miR-125b expression in transfected HeLa cells. HeLa cells were transfected with indicated miRNAs whose over-expression was analyzed by northern blot. Control represent Hela with empty plasmid. Small nuclear U6 (snU6) was used as internal control. 1 representative experiment out of 3 is shown. Intensities of miR-23a and miR-125b bands, alone or in combination, were measured and expressed as arbitrary units on the right of the panel. Values are normalized to snU6 and expressed as mean ± SD of 3 independent experiments.
