# Supplementary material for: IFN-α Regulates Blimp-1 Expression via miR-23a and miR-125b in Both Monocytes-Derived DC and pDC
Source: PLoS One. 2013 Aug 16;8(8):e72833. doi: 10.1371/journal.pone.0072833 (PMC3745402; doi:10.1371/journal.pone.0072833)
Supplement: Figure S3 — Phenotypic, molecular and functional features of IL-4 dC.A. Flow cytometry analysis of lineage-DC markers in IL-4 DC and GM-CSF-treated monocytes. Broken line histograms represent isotype controls. Representative data of 1 experiment out of 3 are shown. B. Expression of pDC–related molecular markers evaluated by qRT-PCR in the same DC populations indicated in panel A. The data are presented as the means ± SD of 3 independent experiments. C. Production of IFN-I in IL-4 DC and pDC after in vitro infection with NDV. DC populations were infected with NDV for 1 hour. Virus was then washed out and the supernatant was harvested after 18 hour incubation and assayed for IFN-I bioactivity, as described in Materials and Methods. Data are representative of 2 independent experiments. Statistical analyses were performed by using Mann-Whitney test (*p≤0.0001, ns = not significant). (PPT) [file pone.0072833.s007.ppt]

## Slide 1
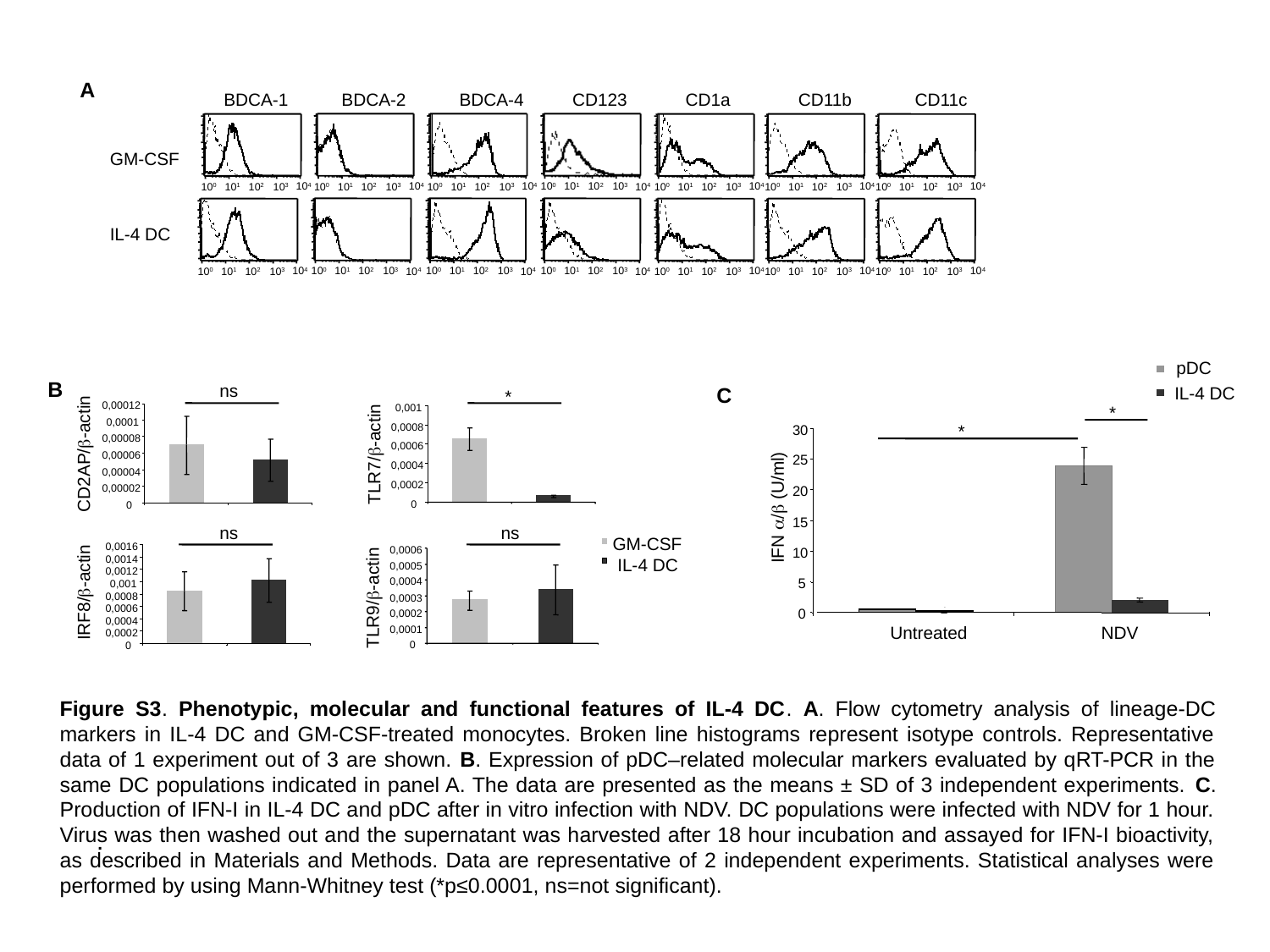

A
BDCA-1 BDCA-2 BDCA-4 CD123 CD1a CD11b CD11c
100
101
102
103
104
104
100
101
102
103
104
100
101
102
103
104
100
101
102
103
104
100
101
102
103
104
100
101
102
103
104
100
101
102
103
100
101
102
103
104
100
101
102
103
104
100
101
102
103
104
104
100
101
102
103
104
100
101
102
103
104
100
101
102
103
104
100
101
102
103
GM-CSF
IL-4 DC
pDC
B
ns
C
*
IL-4 DC
*
0,00012
0,0001
0,00008
0,00006
0,00004
0,00002
0
0,001
*
0,0008
30
0,0006
CD2AP/β-actin
TLR7/β-actin
25
0,0004
0,0002
20
IFN  (U/ml)
0
15
ns
ns
GM-CSF
 IL-4 DC
0,0016
0,0006
10
0,0014
0,0005
0,0012
5
0,0004
0,001
IRF8/β-actin
TLR9/β-actin
0,0008
0,0003
0,0006
0
0,0002
0,0004
Untreated
NDV
0,0001
0,0002
0
0
Figure S3. Phenotypic, molecular and functional features of IL-4 DC. A. Flow cytometry analysis of lineage-DC markers in IL-4 DC and GM-CSF-treated monocytes. Broken line histograms represent isotype controls. Representative data of 1 experiment out of 3 are shown. B. Expression of pDC–related molecular markers evaluated by qRT-PCR in the same DC populations indicated in panel A. The data are presented as the means ± SD of 3 independent experiments. C. Production of IFN-I in IL-4 DC and pDC after in vitro infection with NDV. DC populations were infected with NDV for 1 hour. Virus was then washed out and the supernatant was harvested after 18 hour incubation and assayed for IFN-I bioactivity, as described in Materials and Methods. Data are representative of 2 independent experiments. Statistical analyses were performed by using Mann-Whitney test (*p≤0.0001, ns=not significant).
.
